# Disulfiram ameliorates nonalcoholic steatohepatitis by modulating the gut microbiota and bile acid metabolism

**DOI:** 10.1038/s41467-022-34671-1

**Published:** 2022-11-11

**Authors:** Yuanyuan Lei, Li Tang, Qiao Chen, Lingyi Wu, Wei He, Dianji Tu, Sumin Wang, Yuyang Chen, Shuang Liu, Zhuo Xie, Hong Wei, Shiming Yang, Bo Tang

**Affiliations:** 1 Department of Gastroenterology, Second Affiliated Hospital, Army Medical University, 400037 Chongqing, China; 2 Laboratory Medicine Center, Second Affiliated Hospital, Army Medical University, 400037 Chongqing, China; 3 Jinfeng Laboratory, 401329 Chongqing, China; 4 Department of Laboratory Animal Science, College of Basic Medical Sciences, Army Medical University, 400038 Chongqing, China; 5 Chongqing Institute for Brain and Intelligence, Guangyang Bay Laboratory, 400064 Chongqing, China; 6 Chongqing Municipality Clinical Research Center for Gastroenterology, 400037 Chongqing, China

**Keywords:** Non-alcoholic steatohepatitis, Translational research, Applied microbiology

## Abstract

Nonalcoholic steatohepatitis (NASH) has been linked with the gut-liver axis. Here, we investigate the potential for repurposing disulfiram (DSF), a drug commonly used to treat chronic alcoholism, for NASH. Using a mouse model, we show that DSF ameliorates NASH in a gut microbiota-dependent manner. DSF modulates the gut microbiota and directly inhibits the growth of *Clostridium*. Administration of *Clostridium* abolishes the ameliorating effects of DSF on NASH. Mechanistically, DSF reduces *Clostridium*-mediated 7α-dehydroxylation activity to suppress secondary bile acid biosynthesis, which in turn activates hepatic farnesoid X receptor signaling to ameliorate NASH. To assess the effect of DSF on human gut microbiota, we performed a self-controlled clinical trial (ChiCTR2100048035), including 23 healthy volunteers who received 250 mg-qd DSF for 7 days. The primary objective outcomes were to assess the effects of the intervention on the diversity, composition and functional profile of gut microbiota. The pilot study shows that DSF also reduces *Clostridium*-mediated 7α-dehydroxylation activity. All volunteers tolerated DSF well and there were no serious adverse events in the 7-day follow-up period. Transferring fecal microbiota obtained from DSF-treated humans into germ-free mice ameliorates NASH. Collectively, the observations of similar ameliorating effects of DSF on mice and humans suggest that DSF ameliorates NASH by modulating the gut microbiota and bile acid metabolism.

## Abstract (web summary)

Nonalcoholic steatohepatitis (NASH) has been linked with the gut-liver axis. Here, the authors show that disulfiram (DSF) reduces *Clostridium*-mediated 7α-dehydroxylation activity to suppress secondary bile acid biosynthesis and ameliorate NASH in mice, and validate DSF regulation of the gut-liver axis in healthy men in a self-controlled clinical trial.

## Introduction

Nonalcoholic steatohepatitis (NASH) is the advanced stage of nonalcoholic fatty liver disease (NAFLD)^1^. NASH is characterized by hepatic steatosis (greater than 5%), lobular inflammation, ballooning degeneration of hepatocytes and varying degrees of hepatic fibrosis^2^. The long-term outcomes of NASH are liver cirrhosis and hepatocellular carcinoma^3^. NASH has become a leading cause of liver failure-related transplantation^4^. In addition, NASH also increases non-liver-related serious consequences such as cardiovascular diseases and other malignant tumors^4,5^. To date, there are no approved therapies for NASH^6^. Given the poor prognosis of NASH, finding effective drugs and elucidating its mechanism are unmet clinical needs.

As a method of identifying new applications for existing drugs, drug repurposing is attracting increasing attention. Disulfiram (DSF), also known by its trade name Antabuse, has been approved by the Food and Drug Administration (FDA) and has been used for many years to treat chronic alcoholism by inhibiting the enzyme aldehyde dehydrogenase (ALDH)^7^. Previous studies have shown that DSF has antitumor^7–9^, antiparasitic^10^, and anti-hepatitis C virus infection effects^11^. The efficacy of DSF to treat cocaine abuse^12^, coronavirus disease 2019 (COVID-19)^13^, latent human immunodeficiency virus (HIV) infection^14^ and other conditions has also been widely investigated in clinical trials (https://clinicaltrials.gov). These studies suggest that DSF is a multitarget drug and has potential therapeutic effects on a variety of diseases. Importantly, DSF or its major metabolite has been repeatedly reported by independent groups to exert beneficial effects on metabolic dysfunctions^15–17^. However, in-depth mechanistic research and proper clinical trials of DSF on NASH have not yet been systematically performed.

The liver is exposed to the gut microbiota and its metabolites through the portal vein. Considerable advances in the gut microbiota have improved our understanding of NASH pathogenesis^18^. Furthermore, the gut microbiota has become increasingly recognized as being involved in the treatment of NASH^19^. DSF is an orally administered drug. Thus, it is possible that its benefits for the liver might be due in part to actions in the gut or modulation of the gut microbiota. However, it remains unclear whether DSF ameliorates NASH by modulating the gut microbiota.

Herein, we used a choline‐deficient, L‐amino acid‐defined, high‐fat diet (CDAHFD) to induce a NASH mouse model. We then used antibiotic cocktail (Abx) treatment, fecal microbiota transplantation (FMT) and a human microbiota-associated (HMA) mouse model to demonstrate that DSF ameliorated NASH in a gut microbiota-dependent manner. Furthermore, we analyzed the changes in gut microbiota by assessing 16S ribosomal RNA (rRNA) gene sequencing, metagenomic sequencing and metabolomic analyses. We also conducted a self-controlled clinical trial that demonstrated the potential of DSF to improve blood lipid metabolism and reduce *Clostridium*-mediated 7α-dehydroxylation activity in humans. Finally, transferring fecal microbiota obtained from DSF-treated humans into germ-free (GF) mice ameliorated NASH. We propose a novel role of DSF in ameliorating NASH that involves the gut microbiota and microbial metabolites.

## Results

### Bacterial depletion ablates the ameliorating effects of DSF on NASH

First, wild-type (WT) mice were randomized into control, control+DSF, NASH and NASH + DSF groups and a CDAHFD-induced NASH model was established (Supplementary Fig. 1a). A control+DSF group was used to exclude possible toxic effects of DSF itself. Compared with the NASH mice, serum ALT, AST and hepatic triglyceride (TG) levels were significantly decreased in NASH + DSF mice (Supplementary Fig. 1b–d). In addition, the liver index (%) and a series of liver stains, including hematoxylin and eosin (H&E), oil red O (ORO), α-smooth muscle actin (α-SMA) immunohistochemistry (IHC), Masson and F4/80 immunofluorescence (IF) stainings, indicated that NASH mice typically had hepatic steatosis, lobular inflammation and hepatic fibrosis (Supplementary Fig. 1e). Compared with the NASH mice, a lower NAFLD activity score (NAS), fold change in ORO area, α-SMA-positive area (%), collagen volume fraction (CVF) (%) and F4/80-positive cells (%) were observed in the NASH + DSF mice (Supplementary Fig. 1f). We also tested the mRNA expression of hepatic inflammation- and fibrosis-related indicators by Quantitative RT–PCR (qRT–PCR). We found that DSF significantly reduced the expression of Tnf-α, Mcp-1, Col1α1, Col1α2 and Tgf-β1 (Supplementary Fig. 1g). These results suggest that DSF has potential ameliorating effects on NASH.

**Fig. 1:**
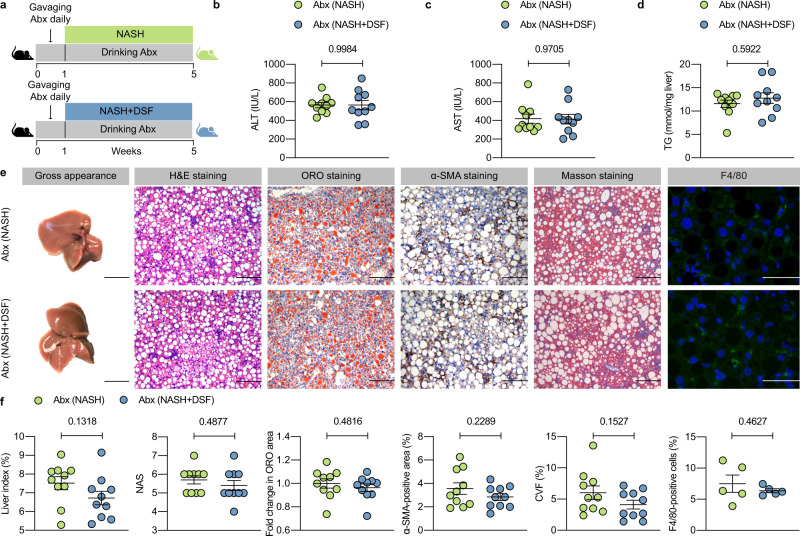
Bacterial depletion ablates the ameliorating effects of DSF on NASH. **a** Abx experimental design. WT mice were put on a course of intragastrically Abx administration for 1 week and received an Abx in the drinking water for another 4 weeks. During the 4 weeks, mice were fed a CDAHFD or CDAHFD + DSF. **b**–**d** Serum ALT and AST; hepatic TG. **e** Representative images of gross appearance of the liver histology (1 cm) and photomicrographs of fixed liver sections after staining with H&E (200 μm), ORO (200 μm), α-SMA antibody (200 μm), Masson (200 μm) and F4/80 antibody (50 μm). **f** Quantification of the liver index (%), NAS, fold change in ORO area, α-SMA-positive area (%), CVF (%) and F4/80-positive cells (%). **a**–**f** *n* = 5 individuals/group for the F4/80 IF staining; *n* = 10 individuals/group for other experiments. Each point represented an individual mouse. Differences in data were calculated by two-sided Mann–Whitney test or unpaired, two-sided *t* test depending on the sample distribution type. Data were represented as mean ± SEM. Exact *P* values were all given. Data were pooled from three independent experiments. Abx antibiotic cocktail, α-SMA α-smooth muscle actin, CVF collagen volume fraction, DSF disulfiram, H&E hematoxylin and eosin, NAS NAFLD activity score, NASH nonalcoholic steatohepatitis, ORO oil red O, TG triglyceride. Source data are provided as a Source Data file.

To identify whether the ameliorating role of DSF was dependent on gut microbiota, an Abx was added to drinking water to deplete gut microbiota (Fig. 1a). The results showed that Abx (NASH) and Abx (NASH + DSF) mice had indistinguishable levels of serum ALT, serum AST, hepatic TG, liver index (%), hepatic steatosis, lobular inflammation, hepatic fibrosis and the mRNA expression of hepatic inflammation- and fibrosis-related indicators (Fig. 1b–f and Supplementary Fig. 2a–e). These results indicate that bacterial depletion ablates the ameliorating effects of DSF on NASH.

### DSF ameliorates NASH in a gut microbiota-dependent manner

We next performed FMT from NASH or NASH + DSF mice to CDAHFD-fed mice, which were denoted as “NASH Recip” or “(NASH + DSF) Recip” mice, respectively (Supplementary Fig. 3a). Compared with NASH Recip mice, the (NASH + DSF) Recip mice showed significantly reduced levels of serum ALT, serum AST, hepatic TG, liver index (%), hepatic steatosis, lobular inflammation and hepatic fibrosis (Supplementary Fig. 3b–f). Consistent with this, FMT from NASH + DSF mice resulted in decreased mRNA expression of hepatic inflammation- and fibrosis-related indicators compared with that from NASH mice (Supplementary Fig. 3g). These data demonstrate that transferring fecal microbiota from DSF-treated mice ameliorates NASH.

**Fig. 2:**
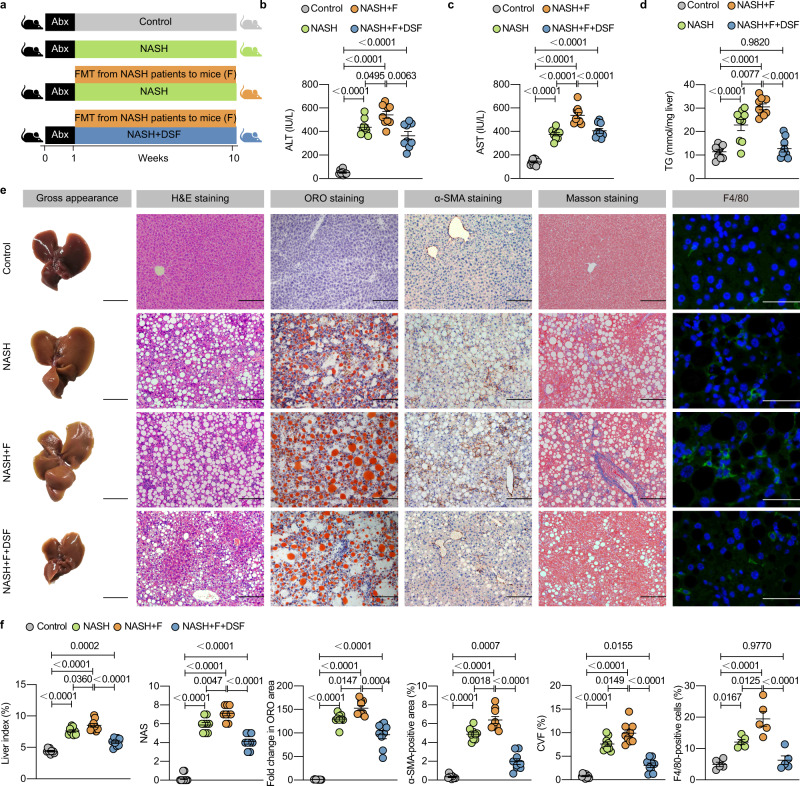
DSF has potential ameliorating effects on mice humanized with the fecal microbiota from NASH patients. **a** HMA experimental design. WT mice were put on a course of intragastrically Abx administration for 1 week and then randomized into 4 groups (control, NASH, NASH + F and NASH + F + DSF) under a control diet, CDAHFD, CDAHFD and CDFHFD + DSF, respectively for 9 weeks. NASH + F and NASH + F + DSF mice were colonized with two NASH patients-derived fecal microbiota for 9 weeks to work in the contexts of the human microbiota. **b**–**d** Serum ALT and AST; hepatic TG. **e** Representative images of gross appearance of the liver histology (1 cm) and photomicrographs of fixed liver sections after staining with H&E (200 μm), ORO (200 μm), α-SMA antibody (200 μm), Masson (200 μm) and F4/80 antibody (50 μm). **f** Quantification of the liver index (%), NAS, fold change in ORO area, α-SMA-positive area (%), CVF (%) and F4/80-positive cells (%). **a**–**f** *n* = 5 individuals/group for the F4/80 IF staining; *n* = 9 individuals/group for other experiments. Each point represented an individual mouse. Differences in data were calculated by Kruskal–Wallis test or ordinary one-way ANOVA depending on the sample distribution type. Data were represented as mean ± SEM. Exact *P* values were all given. Data were pooled from three independent experiments. Abx antibiotic cocktail, α-SMA α-smooth muscle actin, CVF collagen volume fraction, DSF disulfiram, F fecal samples obtained from patients with NASH, FMT fecal microbiota transplantation, H&E hematoxylin and eosin, NAS NAFLD activity score, NASH nonalcoholic steatohepatitis, ORO oil red O, TG triglyceride. Source data are provided as a Source Data file.

To replicate the gut microbiota patterns in NASH patients, we next established an HMA mouse model. We transferred fecal microbiota obtained from NASH patients to microbiota-depleted mice. Mice fed a CDAHFD or CDAHFD + DSF were called “NASH + F” and “NASH + F + DSF” mice, respectively (Fig. 2a). After microbiota transplantation, the levels of serum ALT, serum AST, hepatic TG, liver index (%), hepatic steatosis, inflammation and fibrosis in NASH + F mice were much higher than those in NASH mice. Importantly, the NASH + F + DSF mice exhibited a significantly lower NASH phenotype than NASH + F mice (Fig. 2b–f). Taken together, these data demonstrate that DSF ameliorates NASH in a gut microbiota-dependent manner.

### DSF modulates the gut microbiota and directly inhibits the growth of *Clostridium*

**Fig. 3:**
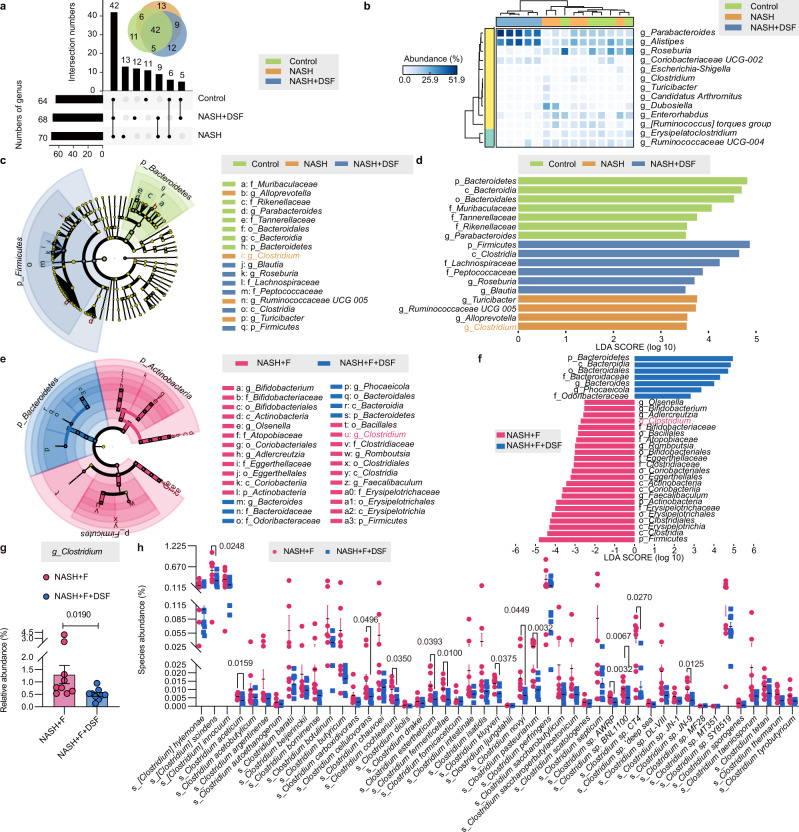
DSF modulates the gut microbiota and apparently reduces the abundance of *Clostridium*. **a**–**d** 16S rRNA gene sequencing analysis in fecal bacterial DNA from control, NASH and NASH + DSF mice was performed. n = 5 individuals/group. **a** In genus level, Upset and Venn diagram of the ASVs in control, NASH and NASH + DSF mice. **b** Heatmap of selected differentially abundant features of control, NASH and NASH + DSF mice (FDR-adjusted *P* < 0.05, LinDA). **c**, **d** Cladograms generated by LEfSe depicting taxonomic association between microbiome communities from control, NASH and NASH + DSF mice (LDA > 3.5). Green, orange and blue bars indicate taxa predominant in control, NASH and NASH + DSF mice, respectively. **e**–**h** Metagenomic sequencing analysis in fecal bacterial DNA from NASH + F and NASH + F + DSF mice was performed. *n* = 9 individuals/group. **e**, **f** Cladograms generated by LEfSe depicting taxonomic association between microbiome communities from NASH + F and NASH + F + DSF mice (LDA > 2.5). Pink bars indicate taxa predominant in NASH + F mice. Blue bars indicate taxa predominant in NASH + F + DSF mice. **g** The abundance of the genus *Clostridium* between NASH + F and NASH + F + DSF mice. FDR-adjusted *P*-value calculated by LinDA was shown. **h** Different species abundance of *Clostridium* in NASH + F and NASH + F + DSF mice. FDR-adjusted *P*-values calculated by LinDA were shown. **a**–**h** Each point represented an individual mouse. Data were represented as mean ± SEM. DSF disulfiram, F fecal samples obtained from patients with NASH, LDA linear discriminant analysis, NASH nonalcoholic steatohepatitis. Source data are provided as a Source Data file.

Considering the role of gut microbiota observed in mice, we hypothesized that DSF treatment acted on the gut microbiota. To investigate this, we carried out 16S rRNA gene sequencing analysis of fecal samples to study differences in gut microbiota among control, NASH and NASH + DSF mice. First, we performed the principal coordinate analysis (PCoA) using Bray–Curtis, Generalized UniFrac, Jaccard, Weighted UniFrac, Unweighted UniFrac and Weighted Normalized UniFrac metric distances (Supplementary Fig. 4a–f), which all manifested that the gut microbiota from these three groups were entirely separated (*P* = 0.001, *P* = 0.002, *P* = 0.001, *P* = 0.003, *P* = 0.001, and *P* = 0.002, respectively). These results indicated that these communities differed in gut microbial composition. Upset and Venn diagrams indicated that 42 shared amplicon sequence variants (ASVs) were present in all groups, whereas 12 ASVs were only present in NASH + DSF mice (Fig. 3a). The annotated heatmap revealed the selected most differentially abundant genera from different groups (Fig. 3b, false discovery rate (FDR)-adjusted *P* < 0.05, LinDA^20^). Furthermore, the linear discriminant analysis (LDA) effect size (LEfSe)^21^ identified several genera that discriminated these groups (LDA score > 3.5). The genus *Parabacteroides* (4.78%, 1.72% and 1.72% in control, NASH and NASH + DSF mice, respectively) was pronouncedly predominant in the control group; the genera *Turicibacter* (0.00%, 0.55% and 0.00%, respectively), *Ruminococcaceae_UCG_005* (0.57%, 0.79% and 0.09%, respectively), *Alloprevotella* (0.71%, 0.83% and 0.06%, respectively) and *Clostridium* (0.01%, 0.60% and 0.00%, respectively) were notably predominant in the NASH group, whereas *Roseburia* (0.17%, 1.70% and 3.91%, respectively) and *Blautia* (1.21%, 3.21 and 4.25%, respectively) were notably predominant in the NASH + DSF group (Fig. 3c, d).

To better understand the effect of DSF on the gut microbiota patterns observed in HMA mice, metagenomic sequencing of fecal samples derived from NASH + F and NASH + F + DSF mice was performed. Alpha diversity indices were measured using different methodologies (ACE, Chao1, Fisher alpha, Observed OTUs and Simpson indices) (Supplementary Fig. 5a). There was no significant difference in alpha diversity between NASH + F and NASH + F + DSF mice (*P* > 0.9999, *P* > 0.9999, *P* = 0.8633, *P* = 0.8633 and *P* = 0.0503 for each index). Then beta diversity analysis of PCoA was performed using Bray–Curtis and Jaccard metric distances which suggested that NASH + F and NASH + F + DSF mice had large differences in their gut microbiota compositions (*P* = 0.003 and *P* = 0.029 for each analysis) (Supplementary Fig. 5b). Moreover, consistent with the 16S rRNA gene sequencing results, these changes also included a marked predominance in *Clostridium* in the NASH + F mice compared with the NASH + F + DSF mice (LDA score > 2.5) (Fig. 3e, f), which were 1.30% and 0.50%, respectively (Fig. 3g, FDR-adjusted *P* = 0.0190, LinDA). Furthermore, we found that DSF treatment significantly reduced the abundance of the *Clostridium* species (13/47). Specially, the abundance of *Clostridium. scindens* (*C. scindens*) which is commonly found in mice and humans^22^ was significantly reduced after DSF treatment (0.57% vs. 0.31%) (Fig. 3h, FDR-adjusted *P* = 0.0248, LinDA). We also confirmed the abundance of *Clostridium* in FMT and HMA mice by qRT–PCR using primers specific for *C. scindens*. The results showed that transferring fecal microbiota from DSF-treated mice significantly reduced *Clostridium* abundance in FMT (Supplementary Fig. 6a) and NASH patients-derived fecal microbiota also significantly increased *Clostridium* abundance in HMA mice (Supplementary Fig. 6b). Collectively, these data suggest that DSF modulates the gut microbiota and apparently reduces the abundance of *Clostridium*.

To further clarify whether *Clostridium* participated in the DSF-induced amelioration of NASH, mice were colonized with *C. scindens* and the colonization of *C. scindens* was confirmed by qRT–PCR (Supplementary Fig. 6c). We found that, surprisingly, compared with NASH + vehicle mice, administration of *Clostridium* alone could exacerbate NASH. Moreover, compared with NASH + DSF + vehicle mice, NASH + DSF + *Clostridium* mice had higher levels of NASH phenotype indexes (Supplementary Fig. 7a–f). Altogether, these data reveal that *Clostridium* abolishes the ameliorating effects of DSF on NASH.

To test the effect of DSF on the growth of *Clostridium*, we added dimethyl sulfoxide (DMSO) or DSF to single culture of *C. scindens*. The in vitro analysis implied that compared with DMSO group, DSF directly inhibited the growth of *C. scindens* (Supplementary Fig. 8a). Then a comparative transcriptomes analysis was carried out to illustrate the role of DSF. The Principal Component Analysis (PCA) result confirmed that compared with DMSO group, DSF treatment resulted in totally different gene expression profiles of *C. scindens* (Supplementary Fig. 8b). Volcano plots displayed that 110 genes were upregulated and 84 genes were down-regulated after DSF treatment (Supplementary Fig. 8c). The Kyoto Encyclopedia of Genes and Genomes (KEGG) enrichment analysis revealed that DSF treatment increased homologous recombination in *C. scindens* (Supplementary Fig. 8d). Additionally, the Gene Ontology (GO)^23^ analysis revealed DSF treatment improved 10 molecular functions including carbohydrate derivative binding, ribonucleotide binding, etc. whereas decreased biological processes related to cellular macromolecule metabolic process and macromolecule metabolic process (Supplementary Fig. 8e, f). These results indicate that DSF directly inhibits the growth of *Clostridium*.

### DSF reduces *Clostridium*-mediated 7α-dehydroxylation activity to suppress secondary bile acid biosynthesis

**Fig. 4:**
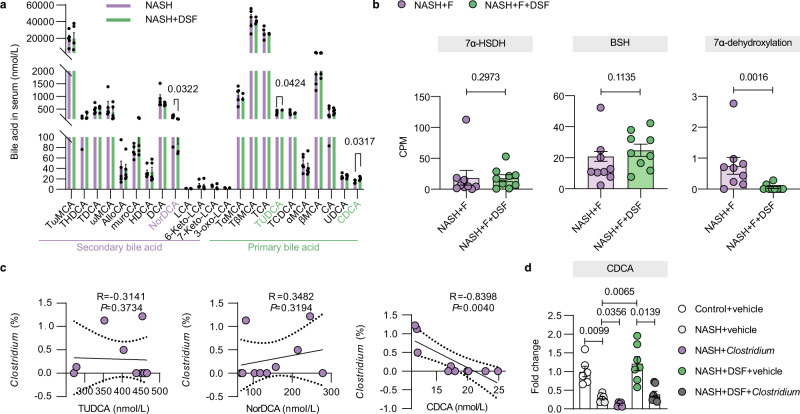
DSF reduces *Clostridium*-mediated 7α-dehydroxylation activity to suppress secondary bile acid biosynthesis. **a** Bile acid levels in the serum between NASH and NASH + DSF groups. n = 5 individuals/group. **b** BSH activity, 7α-HSDH activity and 7α-dehydroxylation activity in NASH + F and NASH + F + DSF groups were profiled by EC number annotations. *n* = 9 individuals/group. **c** The correlations between the abundance of *Clostridium* and TUDCA, NorDCA and CDCA concentrations were analyzed using Spearman’s correlation (two-sided). 5 mice each in NASH and NASH + DSF group were included in the statistics. **d** Fold change of the CDCA level in serum in the administration of *Clostridium* experiment. *n* = 6/6/5/7/7 individuals/group, respectively. **a**–**d** Each point represented an individual mouse. Differences in data between 2 groups were calculated by two-sided Mann–Whitney test or unpaired, two-sided *t* test depending on the sample distribution type. Differences in data for more than 2 groups were calculated by ordinary one-way ANOVA. Data were represented as mean ± SEM. Exact *P* values were all given. CPM counts-per-million, DSF disulfiram, 7α-HSDH hydroxysteroid dehydrogenases, BSH bile salt hydrolase, NASH nonalcoholic steatohepatitis. Source data are provided as a Source Data file.

To clarify the functional consequences of gut microbial changes resulting from DSF treatment, we profiled the gene families and then summed their abundances according to the KEGG database. We found that after DSF treatment, the bacterial functions mainly focused on metabolic regulation (Supplementary Fig. 9a). A previous study has shown that DSF treatment regulates lipid homeostasis and prevents hepatic steatosis in obese mice^15^. Therefore, we further focused on the analysis of lipid metabolism pathways. We found that DSF treatment regulated fatty acid biosynthesis, but short-chain fatty acids (SCFAs) quantification analysis in cecum samples from NASH and NASH + DSF mice showed that SCFAs may not play a role in DSF treatment on NASH (Supplementary Fig. 9b, c). We also found that DSF treatment modulated secondary bile acid biosynthesis (Supplementary Fig. 9b). Therefore, we further profiled quantitative bile acid levels. Compared with NASH mice, the primary bile acids chenodeoxycholic acid (CDCA) and tauroursodeoxycholic acid (TUDCA) in serum were apparently increased (*P* = 0.0317 and 0.0424, respectively), and the secondary bile acid 23-nordeoxycholic acid (NorDCA) was decreased after DSF treatment (*P* = 0.0322) (Fig. 4a). The secondary bile acids level and primary/secondary bile acid ratio in serum and in stool were also evaluated (Supplementary Fig. 10). Similarly, compared with NASH + F mice, the primary bile acids α-muricholic acid (αMCA), βMCA, cholic acid (CA), ursodeoxycholic acid (UDCA) and CDCA levels in serum were significantly increased in NASH + F + DSF mice (*P* = 0.0041, *P* = 0.0262, *P* = 0.0103, *P* = 0.0262 and *P* = 0.0175, respectively), and the secondary bile acid isoallo-lithocholic acid (isoalloLCA) level was apparently decreased after DSF treatment (*P* = 0.0160) (Supplementary Fig. 11). In the gut, primary bile acids are deconjugated and then undergo various microbial reactions, producing abundant secondary bile acids via dehydroxylation, oxidation and epimerization of hydroxyl groups^24^. Then we searched the Enzyme Commission (EC) list of gut microbiota to explore enzyme functions and reaction activities in secondary bile acid biosynthesis. We found that compared with NASH + F mice, DSF treatment did not change the bile acid deconjugation activity of bacteria with bile salt hydrolase (BSH) (*P* = 0.1135) or the hydroxyl oxidation activity (*P* = 0.2973) of bacteria with hydroxysteroid dehydrogenases (HSDHs). However, surprisingly, the 7α-dehydroxylation activity was apparently decreased after DSF treatment (*P* = 0.0016) (Fig. 4b). Bile acid 7α-dehydroxylation is carried out by a small population of anaerobic *Firmicutes*, such as the genus *Clostridium*^25^. Although the abundance of *Clostridium* was not significantly correlated with the TUDCA or NorDCA concentration (*R* = −0.3141, *P* = 0.3734 and *R* = 0.3482, *P* = 0.3194, respectively), a highly significant negative correlation was found between the abundance of *Clostridium* and the primary bile acid CDCA (*R* = −0.8398, *P* = 0.0040) (Fig. 4c). Furthermore, the CDCA level was dramatically decreased after administration of *Clostridium* in vivo (Fig. 4d). Collectively, DSF reduces *Clostridium*-mediated 7α-dehydroxylation activity to suppress secondary bile acid biosynthesis.

### DSF depends on bile acid-induced hepatic farnesoid X receptor (FXR) signaling activation to ameliorate NASH

Bile acids are known to exert their effects primarily through the FXR, which is highly expressed in enterohepatic tissues^26^. Compared with those in NASH Recip mice, noticeable activated FXR signaling in the livers of (NASH + DSF) Recip mice was observed whereas remained unchanged in the intestine (Supplementary Fig. 12a, c, e, f). Moreover, after Abx treatment, the activation of hepatic FXR signaling was no longer observed (Supplementary Fig. 12b, d). These data verify that the gut microbiota is involved in DSF-induced hepatic FXR signaling activation.

**Fig. 5:**
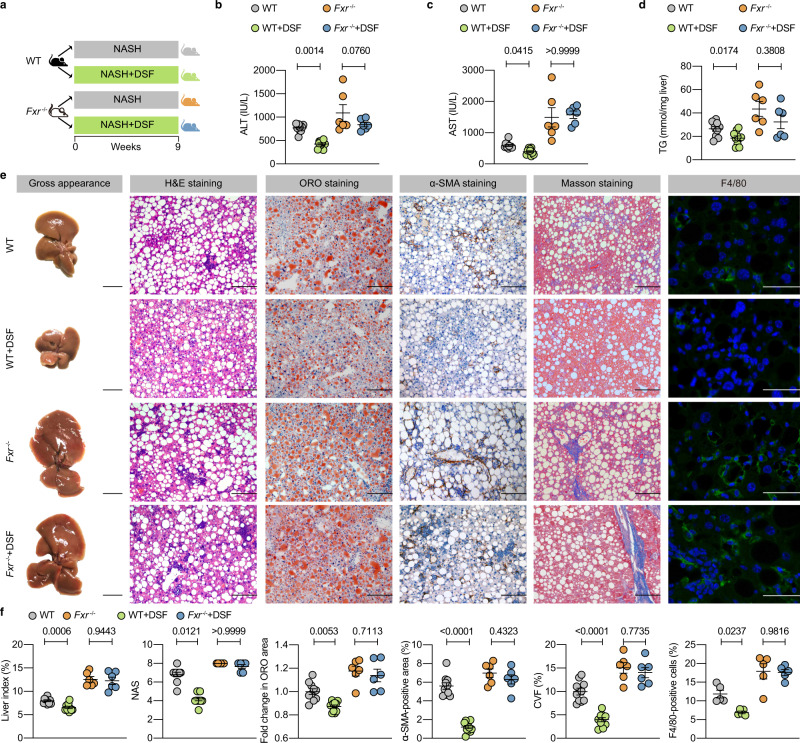
DSF depends on bile acid-induced hepatic FXR signaling activation to ameliorate NASH. **a** Experimental design. WT and *Fxr*^*−/−*^ mice were randomized into 4 groups (WT, WT + DSF, *Fxr*^*−/−*^ and *Fxr*^*−/−*^+DSF) under a CDAHFD or CDAHFD + DSF, respectively for 9 weeks. **b**–**d** Serum ALT and AST; hepatic TG. **e** Representative images of gross appearance of the liver histology (1 cm) and photomicrographs of fixed liver sections after staining with H&E (200 μm), ORO (200 μm), α-SMA antibody (200 μm), Masson (200 μm) and F4/80 antibody (50 μm). **f** Quantification of the liver index (%), NAS, fold change in ORO area, α-SMA-positive area (%), CVF (%) and F4/80-positive cells (%). **a**–**f** *n* = 5 individuals/group for the F4/80 IF staining; *n* = 10/10/6/6 individuals/group, respectively for other experiments. Each point represented an individual mouse. Differences in data were calculated by Kruskal–Wallis test or ordinary one-way ANOVA depending on the sample distribution type. Data were represented as mean ± SEM. Exact *P* values were all given. Data were pooled from three independent experiments. α-SMA α-smooth muscle actin, CVF collagen volume fraction, DSF disulfiram, *Fxr*^*−/−*^ farnesoid X receptor knockout, H&E hematoxylin and eosin, NAS NAFLD activity score, NASH nonalcoholic steatohepatitis, ORO oil red O, TG triglyceride, WT wild-type. Source data are provided as a Source Data file.

It is well established that activating hepatic FXR signaling improves NASH in many ways^26^. To further elucidate the role of FXR signaling in the DSF mechanism of action, we used FXR knockout (*Fxr*^*−/−*^) mice on a CDAHFD and treated them with DSF (Fig. 5a). The results showed that *Fxr*^*−/−*^ mice with or without DSF presented no significant differences in the levels of hepatic steatosis, inflammation or fibrosis, suggesting that the *Fxr*^*−/−*^ mice were unresponsive to DSF (Fig. 5b–f). Thus, we propose that DSF depends on bile acid-induced hepatic FXR signaling activation to ameliorate NASH.

### DSF improves blood lipid metabolism and reduces *Clostridium*-mediated 7α-dehydroxylation activity in humans

**Fig. 6:**
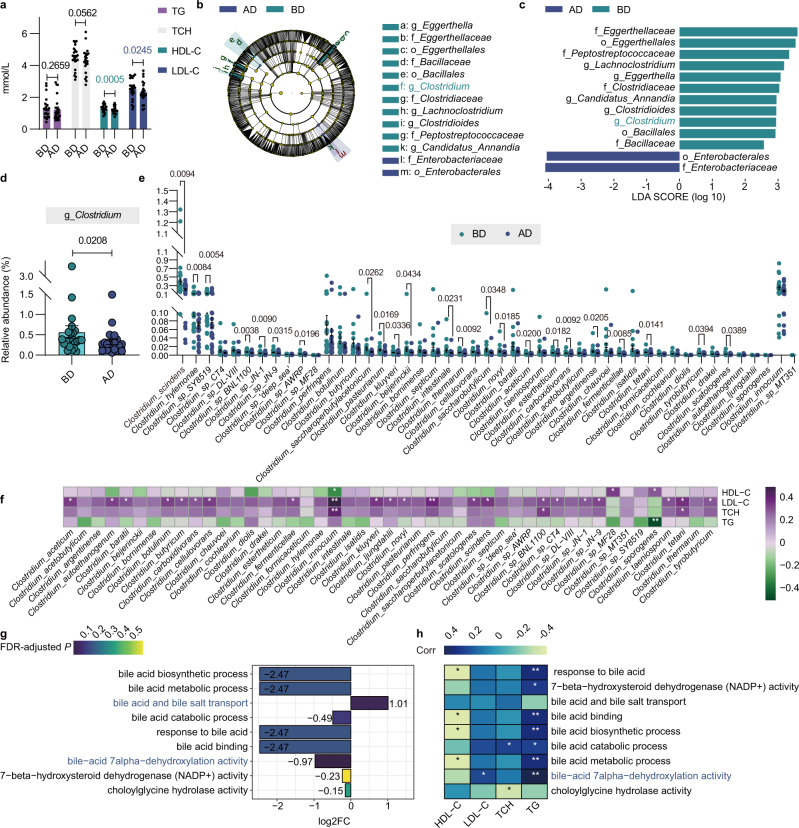
DSF improves blood lipid metabolism and reduces *Clostridium*-mediated 7α-dehydroxylation activity in humans. **a** Blood lipid features including TG, TCH, HDL-C, and LDL-C levels in BD and AD groups. Differences in data between 2 groups were calculated by two-sided paired *t* test. **b**, **c** Cladograms generated by LEfSe depicting taxonomic association between microbiome communities from BD and AD groups (LDA > 2.5). Green and blue bars indicate taxa predominant in BD and AD groups, respectively. **d** Abundance of the genus *Clostridium* between BD and AD groups FDR-adjusted *P*-value calculated by LinDA was shown. **e** Different species abundance of *Clostridium* in BD and AD groups. FDR-adjusted *P*-values calculated by LinDA were shown. **f** Heatmap of the correlations between the species of *Clostridium* and blood lipid features in BD and AD groups. The correlations were analyzed using Spearman’s correlation (two-sided). FDR-adjusted *P* < 0.05 (*P <  0.05 and **P  <  0.01) was shown. **g** Barplot illustrating log2FoldChange relative enrichment of bile acid metabolism activities annotated to the GO function in AD group versus BD group. The color from green to purple represents the significance of the enrichment (two-sided Wilcoxon matched-pairs signed rank test, FDR-adjusted *P* < 0.05). **h** Heatmap of the correlations between bile acid metabolism activities and blood lipid features in BD and AD groups. The correlations were analyzed using Spearman’s correlation (two-sided). FDR-adjusted *P* < 0.05 (*P <  0.05 and **P  <  0.01) was shown. **a**–**h** 23 individuals each in BD and AD groups. Each point represented an individual. Data were represented as mean ± SEM. AD volunteers after DSF treatment, BD volunteers before DSF treatment, HDL-C high-density lipoprotein cholesterol, FDR false discovery rate, LDA linear discriminant analysis, LDL-C low-density lipoprotein cholesterol, TCH total cholesterol, TG triglyceride. Source data are provided as a Source Data file.

Next, we performed a self-controlled clinical trial including 23 healthy volunteers who received 250 mg-qd DSF for 7 days to assess the effect of DSF on the gut microbiota in humans. DSF at a dose of 250 mg daily was shown to be well tolerated in patients^7,12,13^. We collected blood and fecal samples from volunteers before and after DSF treatment, denoted the “BD” and “AD” groups. The primary objective outcomes were to describe the diversity, composition and functional profile of gut microbiota before and after 7 days’ DSF treatment. And the secondary outcomes were to describe the changes in renal function, liver function, blood lipid, blood routine, etc. before and after 7 days’ DSF treatment. DSF did not affect basic characteristics including renal function, liver function, blood routine, etc. in individuals (Supplementary Table 1). Adverse reactions were mainly gastrointestinal symptoms, and there were no serious adverse events in the 7-day follow-up period (Supplementary Table 2). All volunteers tolerated DSF well, and no one dropped out of the trial. Surprisingly, for blood lipid, DSF significantly decreased the levels of high-density and low-density lipoprotein cholesterol (HDL-C and LDL-C) in the participants (*P* = 0.0005 and 0.0245, respectively). Although not reaching statistical significance, the serum TG and total cholesterol (TCH) levels in the AD group were decreased compared with those in the BD group (*P* = 0.2659 and 0.0562, respectively) (Fig. 6a). Then we performed metagenomic sequencing of fecal samples from AD and BD groups. There were no significant differences in gut microbiota alpha diversity and beta diversity between BD group and AD group (*P* = 0.1262, *P* = 0.1262, *P* = 0.1262, *P* = 0.1262 and *P* = 0.6010 for ACE, Chao1, Fisher alpha, Overserved OTUs and Simpson indices; *P* = 0.491 and *P* = 0.836 for Bray–Curtis and Jaccard indices) (Supplementary Fig. 13a, b). But consistent with data observed in mice, LEfSe feature selection identified a marked predominance in *Clostridium* in the BD participants compared with the AD participants (LDA score > 2.5) (Fig. 6b, c). DSF treatment significantly reduced the abundance of the *Clostridium* genus (0.57% vs. 0.33%) and species (23/47) in the human microbiota (Fig. 6d, e, FDR-adjusted *P* < 0.05, LinDA). Most of these species were significantly positively associated with at least one blood lipid feature (FDR-adjusted *P* < 0.05) (Fig. 6f). This is the first report of a substantial decrease in *Clostridium* levels in humans after DSF treatment. We also measured bile acids profile in stool. The results showed that compared with the BD group, the secondary bile acids level was significantly decreased in the AD group (*P* < 0.0001); the primary/secondary bile acid ratio was apparently increased in the AD group (*P* = 0.0059); the secondary bile acids LCA, isoLCA and deoxycholic acid (DCA) were decreased in the AD group (*P* = 0.0004, *P* = 0.0054 and *P* < 0.0001, respectively) (Supplementary Fig. 14a–d). More importantly, the abundance of *Clostridium* was positively correlated with the secondary bile acids level (R = 0.3036, *P* = 0.0402) (Supplementary Fig. 14e).

To predict gut microbiota-mediated bile acid metabolism activity after DSF treatment in humans, we performed GO functional enrichment analysis. Compared with the BD group, the AD group had a notable enrichment of bile acid and bile salt transport activity (log2FC = 1.0137; FDR-adjusted *P* = 0.0301) and a significant lack of bile-acid 7α-dehydroxylation activity (log2FC = 0.9704; FDR-adjusted *P* = 0.0010) (Fig. 6g). The correlation heatmap revealed that bile acid 7α-dehydroxylation activity was positively correlated with serum LDL-C and TG levels (FDR-adjusted *P* = 0.0405 and 0.0001, respectively) (Fig. 6h). These findings indicate that DSF improves blood lipid metabolism and reduces *Clostridium*-mediated 7α-dehydroxylation activity in humans.

### Transferring fecal microbiota obtained from DSF-treated humans into GF mice ameliorates NASH

**Fig. 7:**
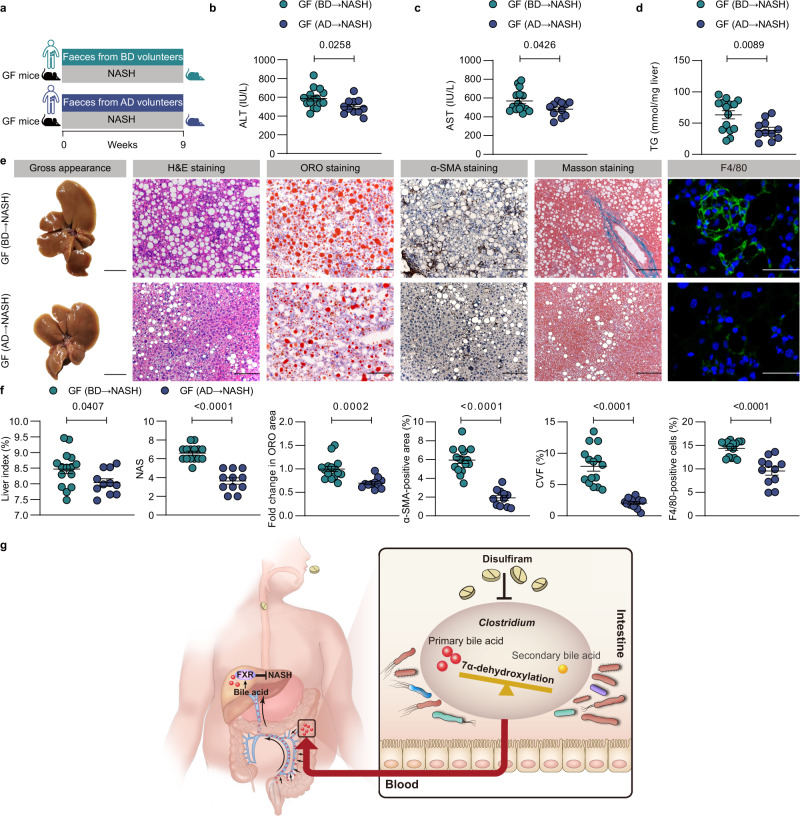
Transferring fecal microbiota obtained from DSF-treated humans into GF mice ameliorates NASH. **a** Experimental design. GF mice were fed a CDAHFD and randomized into 2 groups (GF (BD → NASH) and GF (AD → NASH)). Meanwhile, mice were gavaged with fecal contents obtained from BD and AD humans, respectively twice a week for 9 weeks. **b**–**d** Serum ALT and AST; hepatic TG. **e** Representative images of gross appearance of the liver histology (1 cm) and photomicrographs of fixed liver sections after staining with H&E (200 μm), ORO (200 μm), α-SMA antibody (200 μm), Masson (200 μm) and F4/80 antibody (50 μm). **f** Quantification of the liver index (%), NAS, fold change in ORO area, α-SMA-positive area (%), CVF (%) and F4/80-positive cells (%). **g** Graphical abstract. **a**–**f** *n* = 15/11 individuals/group in BD and AD group, respectively. Each point represented an individual. Differences in data between 2 groups were calculated by two-sided Mann–Whitney test or unpaired, two-sided *t* test depending on the sample distribution type. Data were represented as mean ± SEM. Exact *P* values were all given. Data were pooled from three independent experiments. AD volunteers after DSF treatment, α-SMA α-smooth muscle actin, BD volunteers before DSF treatment, CVF collagen volume fraction, GF germ-free, H&E hematoxylin and eosin, NAS NAFLD activity score, NASH nonalcoholic steatohepatitis, ORO oil red O, TG triglyceride. Source data are provided as a Source Data file.

Finally, we examined the ameliorating effects of DSF-treated and human-derived microbiota on GF mice. GF mice were colonized with the fecal microbiota obtained from BD or AD participants. All mice were fed a CDAHFD to establish NASH (Fig. 7a). As expected, the serum ALT, serum AST, and hepatic TG levels and liver size were dramatically decreased in GF (AD → NASH) mice compared with GF (BD → NASH) mice (Fig. 7b–d). The GF (AD → NASH) mice developed significantly reduced levels of hepatic steatosis, lobular inflammation and hepatic fibrosis (Fig. 7e, f). Moreover, the bile acids profile in serum showed that GF (AD → NASH) mice had a higher primary/secondary bile acid ratio than GF (BD → NASH) mice (*P* = 0.0056). Specifically, compared with GF (BD → NASH) mice, GF (AD → NASH) mice had a higher primary bile acid TCDCA level (*P* = 0.0317) and a lower secondary bile acid DCA level (*P* = 0.0299) (Supplementary Fig. 15). In general, these results indicate that transferring fecal microbiota obtained from DSF-treated humans into GF mice ameliorates NASH.

## Discussion

Herein, we identified a novel role for DSF, which showed potential ameliorating effects on NASH in a gut microbiota-dependent manner. We found that DSF modulated the gut microbial diversity, directly inhibited the growth of *Clostridium* and reduced *Clostridium*-mediated 7α-dehydroxylation activity to suppress secondary bile acid biosynthesis, which in turn activated hepatic FXR signaling to finally ameliorate NASH. We also conducted a self-controlled clinical trial and used GF mice for validation. Overall, the observations of similar ameliorating effects of DSF on mice and humans suggest that DSF is a novel potential gut microbiota-dependent drug for NASH treatment (Fig. 7g).

DSF is an FDA-approved drug used for alcoholism treatment and has been widely recognized as a repurposed and multitargeted drug. Notably, DSF has been repeatedly reported by independent groups to have beneficial effects on metabolic plasticity^15–17^. Supporting these findings, we also confirmed the ameliorating effects of DSF on NASH. The abovementioned studies excluded ALDH inhibition as the mechanism of action of DSF in metabolic plasticity^15^, but unfortunately, the detailed molecular mechanisms of DSF’s effects have not been determined. Disturbances of the gut microbiota in NASH pathogenesis and development have been widely investigated^18,27^. Furthermore, liver-gut axis targets that modulate the gut microbiota and its metabolites are believed to be promising medical therapies for NASH^3,28^. Thus, it is of great interest to identify changes in the gut microbiota and determine DSF-related host targets. We observed that the ameliorating effects of DSF on NASH vanished after gut microbiota depletion. In line with this result, transferring fecal microbiota obtained from DSF-treated mice or humans significantly ameliorated NASH. The HMA mouse model is used to examine the disease phenotypes of bacteria-depleted mice colonized with the fecal microbiota of patients to answer both basic and applied questions in gut microbiota research^29^. Our results observed in HMA mice suggest that DSF also has potential ameliorating effects on mice colonized with the gut microbiota of NASH patients.

Recent research has demonstrated that DSF plus Cu^2+^ modulates gut microbiota in mice with melanoma^30^. However, the role of DSF alone in the gut microbiota remains unclear. In the current study, we observed dramatic shifts in the gut microbial diversities and compositions after DSF treatment in mice and humans. We observed a significant enrichment of *Clostridium* in NASH, a finding that is in agreement with results reported in mouse models of NASH^31,32^. Moreover, a previous study has demonstrated that compared with healthy volunteers, *Clostridium* is enriched in patients with liver cirrhosis, which can progress from NASH^33^. A reduced *Clostridium* abundance has been reported to improve hepatic steatosis and inflammation in NASH mice^34^ and NASH patients^35^. In agreement with these observations, we found that administration of *Clostridium* abolished the ameliorating effects of DSF on NASH, suggesting that *Clostridium* is the key bacterium of NASH group and DSF regulates the NASH phenotype by decreasing *Clostridium*. DSF has previously been revealed to inhibit the growth of various microorganisms thus repurposing it as an antibacterial agent^36–39^. Our data provided new aspects that DSF treatment directly inhibited the growth of *Clostridium*. As we know, a large number of clinically available bactericidal antibiotics target macromolecular biosynthetic processes^40,41^. Consistent with this view, we did observe downregulation of macromolecule metabolic process in *Clostridium* after DSF treatment. Given the above findings, we conclude that a reduction in *Clostridium* is a major contributor to the beneficial effects of DSF in NASH.

The gut microbiota metabolizes hundreds of molecules, such as bile acids, peptidoglycan and SCFAs, many of which influence host physiology and disease. As shown in our previous study, SCFAs play a significant protective role in the mouse caerulein-induced acute pancreatitis model^42^. But in current study, we found that SCFAs may not play a role in DSF treatment on NASH. Notably, bile acids significantly regulate liver homeostasis and have therapeutic potential in NASH^43^. We found that DSF regulated bile acid metabolism and specifically inhibited the microbial 7α-dehydroxylation activity of secondary bile acid biosynthesis. As we known, increasing levels of the primary bile acids cause a dramatic shift toward the *Firmicutes*, particularly *Clostridium cluster XIVa* and increasing production of the harmful secondary bile acid deoxycholic acids. Decreases in *Clostridium cluster XIVa*, which includes bile acid 7α-dehydroxylating bacteria could suppress DCA production^24,25^. In the current study, we observed that the primary bile acids were increased and the secondary bile acids were decreased after DSF treatment. Specially, DSF could reduce *Clostridium*-mediated 7α-dehydroxylation activity to reduce detrimental DCA production. Moreover, the abundance of *Clostridium* was positively correlated with the secondary bile acids level. Thus, our data present an interaction among DSF, bile acids and the abundance of *Clostridium*. Bile acid 7α-dehydroxylation requires a multistep bile acid-inducible (*bai*) regulon-encoded pathway^44^. This multistep *bai*-encoded pathway contains bile acid CoA ligase (*baiB*), bile acid 7α-dehydratase (*baiE*) and so on^25,45^. Thus, it would be of great interest to investigate the detailed distribution of *bai* genes derived from DSF treatment.

Bile acids are known to interact with cellular bile acid receptors. Previous studies have described that activation of FXR favorably regulates glucose homeostasis, lipid metabolism, hepatic inflammation and liver fibrosis in NASH^26^. In addition, FXR has been recognized as a clinically validated approach for treating NASH. Here, we confirmed that *Fxr*^*−/−*^ mice were unresponsive to DSF. Notably, studies have demonstrated that hepatic FXR signaling and intestinal FXR signaling seem to have diametrically opposite effects—activating hepatic FXR prevents steatosis^46^, while intestine-specific *Fxr*^*−/−*^ mice exhibit improved obesity-related metabolic dysfunction^47^. In the current study, we found that DSF activated hepatic FXR signaling rather than intestinal FXR in a gut microbiota-dependent manner.

Globally, dozens of clinical trials have investigated DSF for treating various diseases, including COVID-19, HIV infection, and cocaine abuse (https://www.clinicaltrials.gov/), but none of these studies considered NASH. In agreement with the large-scale use of DSF in previous clinical practice, our clinical trial confirmed that DSF had an acceptable safety profile and was well tolerated in all individuals. Although there are many preclinical candidates for NASH treatment, such as obeticholic acid, aldafermin and GS-0976, they all reportedly increase blood lipid features in humans, which increases the risk of cardiovascular events^48–50^. Surprisingly, we did observe a significant decline in blood lipid features after DSF treatment, suggesting a cardiovascular-protective effect. Additionally, in line with our 16S rRNA gene and metagenomic approach in mice, metagenomic sequencing in humans provided similar information on the composition and functions of the gut microbiota. Thus, our findings help to clarify the possible relationship between DSF and the gut microbiota in the real world. This clinical trial highlights the possibility of DSF for clinical application in NASH. However, the study has some limitations, such as a small sample size and a short treatment duration. Furthermore, it remains unknown how efficacious DSF might be for patients with NASH. Therefore, more robust examinations in larger NASH cohorts are warranted prior to application in clinical practice.

Overall, we describe a mechanism by which DSF modulates the gut microbiota and uses bile acids as messengers to control the amelioration of NASH. These findings not only have possible implications for using DSF as a NASH therapy but also provide a link between gut microbiota, its metabolites and the drug.

## Methods

### A self-controlled clinical trial

The study was in compliance with the CONSORT statement. The research was approved by the Medical Ethics Committee of the Second Affiliated Hospital of Army Medical University (Chongqing, China) (Approved No. 2021-055-01) and was registered with the Chinese Clinical Trial Registry (ChiCTR) in the WHO Registry Network (Registration number: ChiCTR2100048035). The clinical trial was conducted in accordance with the principles of the Declaration of Helsinki. All volunteers provided written informed consent before participated in this study. The first volunteer was included on the 08/01/2021, the last experiment was completed on the 09/01/2021 and the study was finished on the 11/01/2021 after statistical analysis. The manuscript was written in compliance with ICMJE guidelines. Metagenomic sequencing was performed at Novogene in Beijing, China. Bile acid analysis was performed by Metabo-Profile Inc. in Shanghai, China. Other studies were performed in the Second Affiliated Hospital of Army Medical University in Chongqing, China. The clinical trial protocol was also provided.

#### Subjects

Twenty-three healthy volunteers (6 females and 17 males) aged 18–59 years who meet the following exclusion criteria were recruited from the Department of Gastroenterology in the Second Affiliated Hospital of Army Medical University in this study (1: Patients with alcoholic fatty liver disease; 2: Patients with severe renal dysfunction; 3: According to the judgment of the investigators, patients with any clinically significant medical conditions that may affect research participation and/or personal health; 4: Those who have recently taken a variety of drugs for the treatment of diseases; 5: Those who have recently consumed any alcoholic food or used any alcoholic products; 6: Those who are pregnant or breastfeeding; 7: Those who are participated in a weight loss plan within the past 3 months; 8: Those with a weight change of ≥5%; 9: Those who have donated blood within 3 months or plan to donate blood during the study period; 10: Those who have not signed the informed consent form and or could not complete the research process). All volunteers were enrolled by the researchers. Participants didn’t receive cash remuneration. But the research department bore the registration fees and inspection fees before and after drug treatment. The baseline characteristics are presented in Supplementary Table 1.

#### Study procedure and analysis

This study is a self-controlled clinical trial. All subjects received oral DSF (250 mg qd, Mitsubishi Tanabe Pharma Corporation, Osaka, Japan) for 7 days. The height and weight were measured, and the body mass index (BMI) was calculated. Human blood and fecal samples were obtained. Blood samples were obtained after an overnight fast of 10 h, and serum samples was obtained by centrifugation (3000*g*, 10 min). The fecal samples were stored at −80 °C. Clinical parameters including renal function, liver function, blood lipid, blood routine, etc. were determined in the laboratory department of the Second Affiliated Hospital of Army Medical University. Liver stiffness and hepatic fat attenuation were confirmed by a noninvasive, integrated image-guided detection system (the FibroTouch system) to assess the liver fibrosis and fatty liver levels. Adverse reactions that occurred during the 7-day follow-up period were described in Supplementary Table 2.

#### Outcomes

The primary objective outcomes were to describe the diversity, composition and functional profile of gut microbiota before and after 7 days’ DSF treatment.

The secondary outcomes were to describe the changes in renal function, liver function, blood lipid, blood routine, etc. before and after 7 days’ DSF treatment.

### Animals

The animal protocols were approved by the Laboratory Animal Welfare and Ethics Committee and adhered to the Animal Ethics Statement (Approved No. AMUWEC20210143) (Army Medical University). C57BL/6 WT mice were purchased from Vital River Laboratories (Beijing, China). *Fxr*^*−/−*^ mice with a C57BL/6 background were obtained from the Jackson Laboratory (Cat# 007214, Bar Harbor, USA). Prof. W.H. from the Department of Laboratory Animal Science (Army Medical University) provided the GF mice. Male mice weighing 20–22 g and aged 6–8 weeks at the beginning of the study were used. Mice were bred under a specific pathogen-free (SPF) environment with controlled conditions (12-h light/dark cycle at 21 ± 1 °C and 45 ± 5% humidity). Throughout the study, mice were given ad libitum access to food and water. Across all experimental conditions, mice were all of the same species, sex, age and rearing conditions (at Army Medical University) and were strictly randomized into groups.

### NASH model establishment and DSF treatment

To establish the NASH model, mice were fed a CDAHFD (Cat# A06071302, Research Diets, New Brunswick, USA) for 9 weeks. Mice that received an L-amino acid diet with 10 kcal% fat with methionine and choline (Cat# A06071314, Research Diets) served as controls. DSF was obtained from MedChemExpress (Cat# HY-B0240, MCE, New Jersey, USA) and was included in the control diet or CDAHFD at a concentration of 1.667 g DSF/kg diet, which was formulated to provide mice with a daily dose of 250 mg DSF/kg body weight.

### ALT and AST levels

Blood samples were obtained after 10 h of overnight fasting. The serum was obtained by centrifugation (3000*g*, 10 min). The ALT and AST levels were detected by an automatic biochemical analyzer at the Second Affiliated Hospital of Army Medical University.

### TG assay

Liver TG levels were measured using a TG assay kit (Cat# A110-1-1, Njjcbio, Nanjing, China). Briefly, liver tissue was added to an ice-cold 1.5 mL tube, and absolute ethanol was added (liver tissue weight (g): absolute ethanol volume (mL) = 1:9). The tissue was then homogenized and centrifuged (600*g*, 10 min). The supernatant was taken as the sample. Next, 2.5 μL of standard or sample and 250 μL of working fluid were added to a 96-well plate, and the plate was heated for 10 min at 37 °C. The absorbance was detected at 510 nm by a microplate reader (Cat# VL0L00D0, Thermo Scientific, Waltham, USA). Data were represented as mmol/mg liver.

### Liver histology

Liver index (%) was defined as the ratio of liver (g) to body weight (g). For ORO staining, fresh liver was embedded in optimal cutting temperature compound (Cat# 450083, Sakura Finetek, USA). Frozen 10 μm sections were obtained with a cryostat (Leica Biosystems, Cat# CM3050S, Nussloch, Germany) and stained with ORO (Cat# G1261, Solarbio, Beijing, China) following the manufacturer’s instructions.

For H&E staining, IHC staining, Masson staining and IF staining, fresh liver was fixed in 10% neutral-buffered formalin and then embedded in paraffin. Five-micrometer adjacent sections of tissue blocks were used. H&E staining was semi-quantitatively scored using NAS criterion (Supplementary Table 3) by a board-certified veterinary pathologist in a blinded manner. IHC staining was performed using the following antibodies: α-SMA polyclonal antibody (1:1000; Cat# 14395-1-AP, Proteintech, Rosemont, USA), FXR (1:200; Cat# sc-25309, Santa Cruz Biotechnology, Santa Cruz, USA) and anti-CYP7A1 (1:100; Cat# sc-518007, Santa Cruz Biotechnology). IF staining was performed using anti-F4/80 rabbit polyclonal antibody (1:500, Cat# GB11027, Servicebio, Wuhan, China). Images of liver histology were taken with an upright fluorescence microscope (Olympus, BX63, Japan). Quantification was performed using ImageJ software (v1.8.0, National Institutes of Health, NIH; http://www.imagej.softonic.de).

### RNA extraction of tissue and qRT–PCR

Liver and intestine tissues were frozen in liquid nitrogen. A TRizon-RNA Extract kit (Cat# P2002, Engreen, Beijing, China) was used to extract total RNA. The PrimeScript RT reagent kit (Cat# RR047A, Takara, Kusatsu, Japan) was used to synthesize cDNA. qRT–PCR was conducted with a TB Green Premix Ex Taq II kit (Cat# RR820A, Takara). The β-actin gene was used as a control. The primer sequences were summarized in Supplementary Table 4.

### Abx treatment

Mice received a daily oral gavage (200 μL/mouse) of an Abx consisting of metronidazole (200 mg/kg), neomycin sulfate (200 mg/kg), ampicillin (200 mg/kg) and vancomycin (100 mg/kg) for 1 week (Cat# A600633, Cat# A610366, Cat# A610028 and Cat# A600983, Sangon, Shanghai, China). Throughout the next 4 weeks, the mice received an Abx in the drinking water containing neomycin sulfate (1 g/L), metronidazole (1 g/L), ampicillin (1 g/L) and vancomycin (500 mg/L). The antibiotic-containing water was replaced every other day.

### FMT

The feces samples were homogenized in PBS with 20% glycerin in a 50 mL tube (20 mg feces/1 mL liquid). The tube was then centrifuged (100*g*, 10 min), and the whole supernatant was aliquoted and frozen at −80 °C. For FMT in SPF-housed WT mice, the mice were first treated with Abx for 1 week to deplete the gut microbiota. They were then fed a CDAHFD for 9 weeks to establish the NASH model. During the 9 weeks, the mice were randomized into 2 groups (referred to as “NASH Recip” and “(NASH + DSF) Recip”), and twice weekly they were administered orally with feces (suspended in PBS, 200 µL/mouse) derived from CDAHFD-fed mice (referred to as “NASH donors”) and CDAHFD + DSF-fed mice (referred to as “NASH + DSF donors”).

For FMT of GF mice, the GF mice were fed a CDAHFD for 9 weeks to establish the NASH model. During the 9 weeks, the mice were randomized into 2 groups (referred to as “GF (BD → NASH)” and “GF (AD → NASH)”) and were orally administered feces (suspended in PBS, 200 µL/mouse) obtained from BD individuals and AD individuals, respectively.

### HMA mice and human fecal microbiota transfer

For human fecal microbiota transfer, fecal samples were obtained from two patients with NASH (F). The steps of preparing fecal samples were performed as described in the FMT section above. WT mice were first treated with Abx (as described in the Abx treatment section above) for 1 week to deplete the gut microbiota and then were randomized into 4 groups. Group 1: mice were fed a control diet and administered orally with vehicle; Group 2: mice were fed a CDAHFD and administered orally with vehicle; Group 3: mice were fed a CDAHFD and administered orally with the above fecal supernatant; Group 4: mice were fed a CDAHFD + DSF and administered orally with the above fecal supernatant (200 μL/mouse, twice per week for 9 weeks).

### Bacterial culture, detection, transplantation and treatment

*C. scindens* was obtained from American Type Culture Collection (ATCC, Cat# 35704, ATCC, Maryland, USA) and anaerobically cultured in ATCC medium 2107. For bacterial transplantation in vivo, WT mice were first treated with Abx for 1 week to deplete the gut microbiota and then fed a control diet/CDAHFD/CDAHFD + DSF. *C. scindens* was administered orally at 3 × 10^8^ CFU of bacteria/mouse twice per week for 9 weeks. Equal sterile 2107 medium was used as a vehicle. For bacteria treatment in vitro, *C. scindens* was cultured in ATCC medium 2107 (liquid) under anaerobic conditions using an anaerobic chamber (GeneScience, E500, USA) and was treated with DSF (20 μg/mL) or equivalent DMSO (Cat# ST0380, Beyotime, Shanghai, China). After 24 h, absorbance was measured at 600 nm using a microplate reader. Bacteria detection was confirmed by qRT–PCR. The specific primers of Universal 16S (Universal 16S 27F: 5′-AGAGTTTGATCCTGGCTCAG-3′; Universal 16S 1492R: 5′-AAGTCGTAACAAGGTAACC-3′) and *C. scindens* (Scin F1: 5′-GCAACCTGCCTTGCACT-3′; Scin R1: 5′-ACCGAATGGCCTTGCCA-3′) were from previous study^51^.

### Bacterial RNA extraction and comparative transcriptomes analysis

For bacterial RNA extraction, *C. scindens* treated with DMSO and DSF were collected and used for total RNA extraction. Bacteria were preserved in liquid nitrogen. Total RNA was extracted with TRizon-RNA Extract kit (Cat# P2002, Engreen). RNA integrity was assessed using the RNA Nano 6000 Assay Kit of the Bioanalyzer 2100 system (Agilent Technologies, CA, USA).

For bacterial comparative transcriptomes analysis, library preparations were sequenced on an Illumina NovaSeq 6000 platform by Novogene and 150 bp paired-end reads were generated. Raw sequences were quality-filtered and mapped to the *C. scindens* reference genome, available from NCBI Genome database (ID: 977). HTSeq (v0.6.1) was used to count the reads numbers mapped to each gene. And then Fragments Per Kilobase of exon model per Million mapped reads of each gene was calculated based on the length of the gene and reads count mapped to this gene. Then PCA were calculated and visualized. Analysis of similarities (ANOSIM) test was performed to calculate significance of dissimilarity between sample groups. Differential expression analysis of 2 groups was performed using the DESeq R package (v1.18.0). The resulting *P*-values were adjusted using the Benjamini and Hochberg’s approach for controlling the FDR. Genes with an adjusted *P*-value <0.05 found by DESeq were assigned as differentially expressed and a volcano map was drawn. After the differential gene expression analysis, the differentially expressed genes were subjected to GO and KEEG enrichment analysis. Dotplot was employed to visualize the result of functional enrichment analysis.

### Fecal genomic DNA extraction

Fecal samples (100 mg) from humans or mice were used to obtain fecal genomic DNA with the TIANamp Stool DNA kit (Cat# DP328-02, Tiangen, Beijing, China). The DNA purity and concentration were measured with a NanoDrop 2000C spectrophotometer (Thermo Scientific).

### 16S rRNA gene sequencing

The specific primers for the 16S rRNA gene V3-V4 regions were 341F (CCTAYGGGRBGCASCAG) and 806 R (GGACTACNNGGGTATCTAAT). A GeneJET Gel Extraction kit (Cat# K069, Thermo Scientific) was used to conduct rapid and efficient purification of the PCR products. All libraries were sequenced using the Illumina NovaSeq 6000 platform at Novogene. The primers of paired-end fastq format sequences were trimmed by *cutadapt* (v2.8)^52^ with Python (v3.6.7). The trimmed sequencing data were processed by the Quantitative Insights into Microbial Ecology 2 kit (QIIME2, v2020.2)^53^ within a conda environment (miniconda v4.10.3). The trimmed fastq files were imported into QIIME2. The reads were then denoised into ASVs, and a representative sequence (rep-seqs.qza) of each ASV and feature table (table.qza) were generated. After the DADA2^54^ denoising step was completed, a “denoising-stats.qzv” file was generated that listed how many “nonchimeric” sequences were left-associated with each sample after denoising. Samples with a depth of less than 8000 were excluded (nonchimeric sequences). All remaining samples were then rarefied to the same depth, and a rarefied feature table was created. After rarefaction, alpha-diversity was calculated by “qiime diversity alpha” command from the rarefied feature table. The Bray–Curtis, Generalized UniFrac, Jaccard, Weighted UniFrac, Weighted Normalized UniFrac and Unweighted UniFrac metric distances were calculated. After computing the diversity metrics, the PCoA was computed and visualized. The significance of dissimilarity between sample groups was calculated by ANOSIM. Taxonomy was assigned using a pretrained naïve Bayes classifier (silva-132-99-nb-classifier.qza, trained on SILVA^55^ 132 99% full-length sequences). Differences in bacterial abundance were analyzed using linear models for differential abundance analysis (LinDA) and FDR-adjusted *P*-values of differential abundance were provided. Heatmap was generated by Statistical Analysis of Metagenomic Profiles software (STAMP, v2)^56^. The predominance of bacterial communities between groups was analyzed using the LEfSe (LDA score (log10) = 3.5 as cutoff value).

A Phylogenetic Investigation of Communities by Reconstruction of Unobserved States kit (PICRUSt2)^57^ within another conda environment was used to predict functional abundances which were mapped to the KEGG orthologs (KO) and KEGG pathways.

### Metagenomic sequencing

All libraries were sequenced, and reads were generated using the Illumina NovaSeq 6000 platform at Novogene. KneadData software (v0.7.4; http://huttenhower.sph.harvard.edu/kneaddata) was used to process the raw fq.gz sequencing data files. KneadData was run by calling Bowtie2 (v2.4.2)^58^ to remove host genome contamination in the samples. Trimmomatic (v0.39)^59^ was used to remove sequencing primers to obtain clean data. Conda was used to establish the Kraken2 environment and to install Kraken2 (v2.1.1) and Bracken (v2.6.0). The Kraken2^60^ and Bracken^61^ databases were downloaded. Kraken2 software was run to obtain the taxonomic counts in each sample, and Bracken software was used to adjust and convert the counts data into relative abundance. The Bray–Curtis and Jaccard metric distances were calculated. After computing the diversity metrics, the PCoA was computed and visualized. The significance of dissimilarity between sample groups was calculated by ANOSIM. The predominance of bacterial communities between groups was analyzed using the LEfSe (LDA score (log10) = 2.5 as cutoff value). Differences in bacterial abundance were analyzed using LinDA and *P* values were adjusted by Benjamini & Hochberg method to control FDR. FDR-adjusted *P*-values were provided.

Functional annotations were performed by using the data files from the HMP Unified Metabolic Analysis Network 3.0^62^ (HUMAnN 3.0, v3.0.0; https://huttenhower.sph.harvard.edu/humann). The clean paired-end sequencing data were merged into a single fastq file. The HUMAnN 3.0 toolkit was run by using the “humann–input myseq*.fq–output humann3/–threads 32–memory-use maximum -r -v” command, which calls Bowtie2^58^ to compare nucleic acid sequences and calls DIAMOND^63^ to compare protein sequences to complete gene/protein function annotation and to obtain Uniref90^64^ annotation. Next, the UniRef90 table was mapped to the EC and the GO terms. Thus, each protein sequence was annotated with UniRef90, EC, and GO terms (counts-per-million, CPM). “EC1.17.98.1: Bile-acid 7-alpha-dehydroxylase”, “EC3.1.2.26: Bile-acid-CoA hydrolase” and “EC4.2.1.106: Bile-acid 7-alpha-dehydratase” were merged into “Bile-acid 7- alpha-dehydroxylation”. “GO:0033792: [MF] bile-acid 7alpha-dehydroxylase activity”, “GO:0033881: [MF] bile-acid-CoA hydrolase activity” and “GO:0033988: [MF] bile-acid 7alpha-dehydratase activity” were merged into “bile-acid 7alpha-dehydroxylation activity”.

### Bile acid analysis

Briefly, blood samples were obtained after an overnight fast of 10 h, and serum was obtained by centrifugation (3000*g*, 10 min). The fecal samples were stored at −80 °C. Bile acid analysis was performed by Metabo-Profile Inc. using UPLC-MS/MS (ACQUITY UPLC-Xevo TQ-S, Waters Corp., Milford, MA, USA)^65^.

### SCFAs quantification analysis

The concentrations of SFCAs in cecal content were detected using a gas chromatography-mass spectrometry (GC-MS) system (Trace 1300 and ISQ 7000, Thermo Scientific) (BioNovoGene, Suzhou, China). SCFA standards including acetic acid, propionic acid, isobutyric acid, butyric acid, isovaleric acid and valeric acid were purchased from Sigma-Aldrich (St. Louis, USA).

### Statistical analysis

All error bars represent the standard error of the mean (SEM). Statistical significance was assigned at *P* values of <0.05 and detected by GraphPad Prism software (v8, GraphPad Software Inc., San Diego, USA). The Shapiro–Wilk test was used to determine the sample distribution type. For 2 groups, the F test was used to test homoscedasticity, and significance was calculated by the two-sided Mann–Whitney test or unpaired, two-sided *t* test depending on the sample distribution type. For more than 2 groups, the Brown-Forsythe test was used to test homoscedasticity, and the significance was calculated by the Kruskal–Wallis test or ordinary one-way ANOVA depending on the sample distribution type; a post hoc Tukey test was used to conduct multiple comparisons. Multiple testing was corrected using the Benjamini-Hochberg method to control the FDR and FDR-corrected *P* value <0.05 was considered statistically significant. For clinical trial analysis, differences in data between 2 groups were calculated by two-sided Wilcoxon matched-pairs signed rank test or two-sided paired *t* test depending on the sample distribution type. The correlation analysis was investigated by Spearman’s test (two-sided).

### Reporting summary

Further information on research design is available in the Nature Portfolio Reporting Summary linked to this article.

## Supplementary information

Supplementary Information

Reporting Summary

STORMS-checklist

## Data Availability

The comparative transcriptomes, metagenomic and 16S rRNA gene sequencing data generated in this study have been deposited in the European Nucleotide Archive database under accession code PRJEB49623. The raw healthy volunteer-related clinical trial raw data, which could compromise protection of privacy of research participants are protected and are not available due to data privacy laws. The data supporting the findings of this study generated in this study are provided in the Supplementary Information and Source Data file. Source data are provided as a Source Data file. Source data are provided with this paper.

## Source data

Source Data

## Code availability

Custom code supporting the statistical analysis are publicly available at GitHub (10.5281/zenodo.7247972^66^).
