# Supplementary material for: Disulfiram ameliorates nonalcoholic steatohepatitis by modulating the gut microbiota and bile acid metabolism
Source: Nat Commun. 2022 Nov 11;13:6862. doi: 10.1038/s41467-022-34671-1 (PMC9651870; doi:10.1038/s41467-022-34671-1)
Supplement: Supplementary file 1 — Supplementary Information [file 41467_2022_34671_MOESM1_ESM.pdf]

Supplementary Information for

Disulfiram ameliorates nonalcoholic steatohepatitis by modulating the gut microbiota and bile acid metabolism

Yuanyuan Lei[1], Li Tang[1], Qiao Chen[1], Lingyi Wu[1], Wei He[1], Dianji Tu[1, 2], Sumin Wang[1], Yuyang Chen[1], Shuang Liu[1], Zhuo Xie[1], Hong Wei[3, 4*], Shiming Yang[1, 5, 6*], Bo Tang[1*]

[1]Department of Gastroenterology, Second Affiliated Hospital, Army Medical University, Chongqing, 400037, China

[2]Laboratory Medicine Center, Second Affiliated Hospital, Army Medical University, Chongqing, 400037, China

[3]Jinfeng Laboratory, Chongqing, 401329, China

[4]Department of Laboratory Animal Science, College of Basic Medical Sciences, Army Medical University, Chongqing, 400038, China

[5]Chongqing Institute for Brain and Intelligence, Guangyang Bay Laboratory, Chongqing, 400064, China

[6]Chongqing Municipality Clinical Research Center for Gastroenterology, Chongqing, 400037, China

[*]**Correspondence: These authors jointly supervised this work.**

[*]Prof. Hong Wei, PhD.

Jinfeng Laboratory, Chongqing, 401329, China; E-mail: weihong63528@163.com.

[*]Prof. Shiming Yang, MD, PhD.

Department of Gastroenterology, Second Affiliated Hospital, Army Medical University, Chongqing, 400037, China; E-mail: yangshiming@tmmu.edu.cn.

[*]Prof. Bo Tang, MD, PhD.

Department of Gastroenterology, Second Affiliated Hospital, Army Medical University,

Chongqing, 400037, China; E-mail: botangxq@tmmu.edu.cn.

ORCID: https://orcid.org/0000-0001-9900-2150 (To Yuanyuan Lei); https://orcid.org/0000-0001-6069-1067 (To Hong Wei); https://orcid.org/0000-0002-0000-4795 (To Shiming Yang); https://orcid.org/0000-0003-4089-5155 (To Bo Tang)

This PDF file includes:

Supplementary Fig. 1 to Fig. 15

Supplementary Table 1 to Table 4

Supplementary Figures

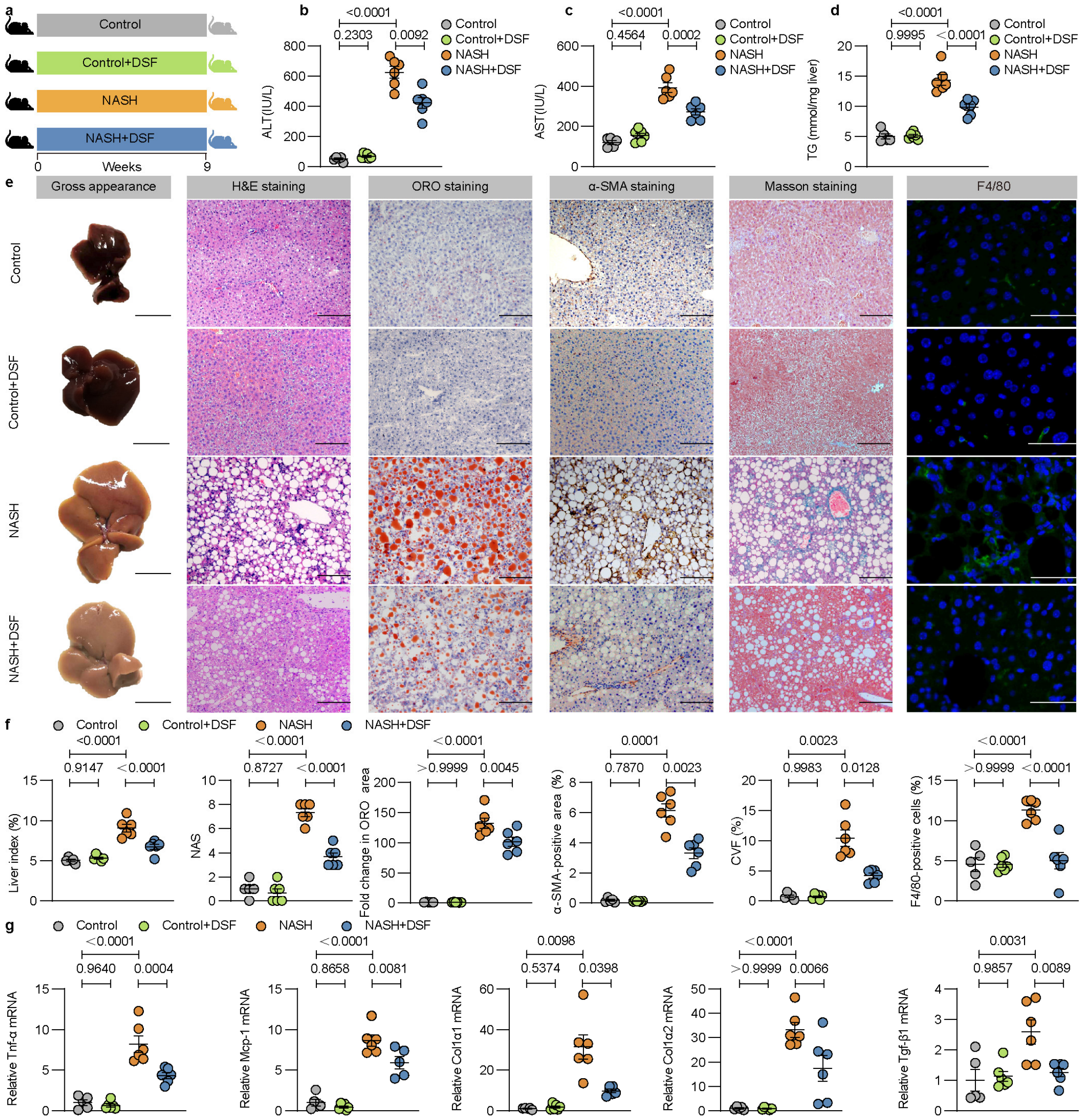

Supplementary Fig. 1. DSF has potential ameliorating effects on NASH. a Experimental design. WT mice were randomized into 4 groups (control, control+DSF, NASH and NASH+DSF) under a control diet, control+DSF diet, CDAHFD and CDFHFD+DSF, respectively for 9 weeks to explore the role of DSF. **b-d** Serum ALT and AST; hepatic TG. **e** Representative images of gross appearance of the liver histology (1 cm) and photomicrographs of fixed liver sections after staining with H&E (200 μm), ORO (200 μm), α-SMA antibody (200 μm), Masson (200 μm) and F4/80 antibody (50 μm). **f** Quantification of the liver index (%), NAS, fold change in ORO

area, α-SMA-positive area (%), CVF (%) and F4/80-positive cells (%). **g** The mRNA expressions of inflammation-related (Tnf-α, Mcp-1) and fibrosis-related (Col1α1, Col1α2 and Tgf-β1) indicators in control, NASH and NASH+DSF groups were confirmed by qRT–PCR. **a-g** n = 5-6 individuals/group. Each point represented an individual mouse. Differences of data were calculated by Kruskal-Wallis test or ordinary one-way ANOVA depending on the sample distribution type. Data were represented as mean ± SEM. Exact *P* values were all given. Data were pooled from three independent experiments. α-SMA, α-smooth muscle actin; CVF, collagen volume fraction; DSF, disulfiram; H&E, hematoxylin and eosin; NAS, NAFLD activity score; NASH, nonalcoholic steatohepatitis; ORO, oil red O; TG, triglyceride. Source data are provided as a Source Data file.

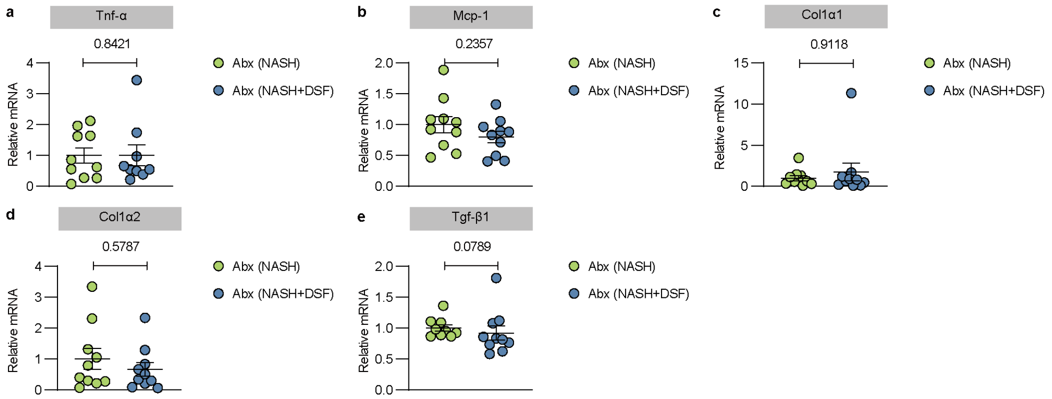

Supplementary Fig. 2. Bacterial depletion ablates the ameliorating effects of DSF on inflammation- and fibrosis-related indicators on NASH. The mRNA expressions of inflammation-related (Tnf-α, Mcp-1) **a-c** and fibrosis-related (Col1α1, Col1α2 and Tgf-β1) **d, e** indicators in Abx (NASH) and Abx (NASH+DSF) were confirmed by qRT–PCR. **a-e** n = 9-10 individuals/group. Each point represented an individual mouse. Differences of data by calculated by two-sided Mann-Whitney test or unpaired, two-sided t test depending on the sample distribution type. Data were represented as mean ± SEM. Exact *P* values were all given. Data were pooled from three independent experiments. Abx, antibiotic cocktail; DSF, disulfiram; NASH, nonalcoholic steatohepatitis. Source data are provided as a Source Data file.

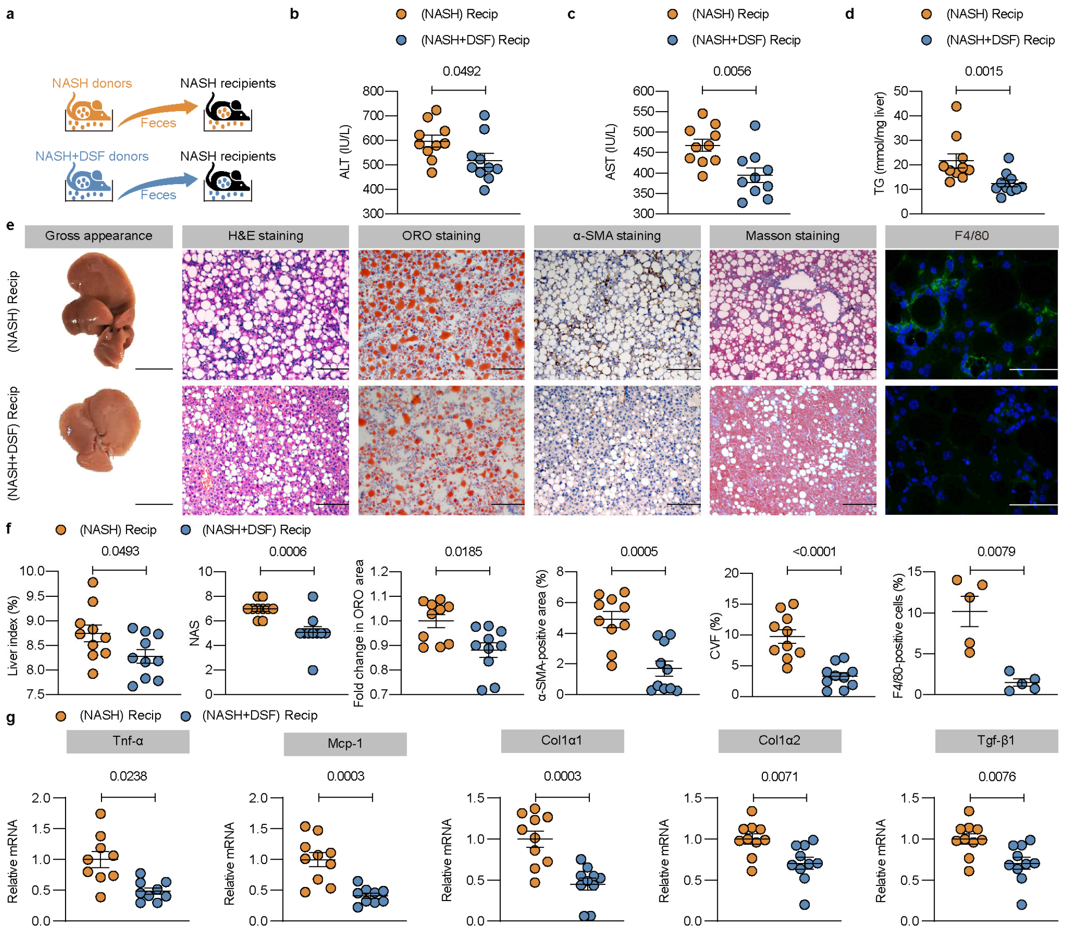

Supplementary Fig. 3. Transferring fecal microbiota from DSF-treated mice ameliorates NASH. a FMT experimental design. WT mice were put on a course of intragastrically Abx administration for 1 week for gut microbiota depletion prior to FMT. During FMT, mice were fed a CDAHFD and gavaged with the fecal contents of either NASH or NASH+DSF donor mice for 9 weeks. **b-d** Serum ALT and AST; hepatic TG. **e** Representative images of gross appearance of the liver histology (1 cm) and photomicrographs of fixed liver sections after staining with H&E (200 μm), ORO (200 μm), α-SMA antibody (200 μm), Masson (200 μm) and F4/80 antibody (50 μm). **f** Quantification of the liver index (%), NAS, fold change in ORO area, α-SMA-positive area (%), CVF (%) and F4/80-positive cells (%). **g** The mRNA expressions of inflammation-related (Tnf-α, Mcp-1) and fibrosis-related (Col1α1, Col1α2 and Tgf-β1) indicators were confirmed by qRT–PCR. **a-g** n = 5 individuals/group for the F4/80 IF

staining; n = 9-10 individuals/group for other experiments. Each point represented an individual mouse. Differences of data by calculated by two-sided Mann-Whitney test or unpaired, two-sided t test depending on the sample distribution type. Data were represented as mean ± SEM. Exact *P* values were all given. Data were pooled from three independent experiments. α-SMA, α-smooth muscle actin; CVF, collagen volume fraction; H&E, hematoxylin and eosin; NAS, NAFLD activity score; NASH, nonalcoholic steatohepatitis; ORO, oil red O; TG, triglyceride. Source data are provided as a Source Data file.

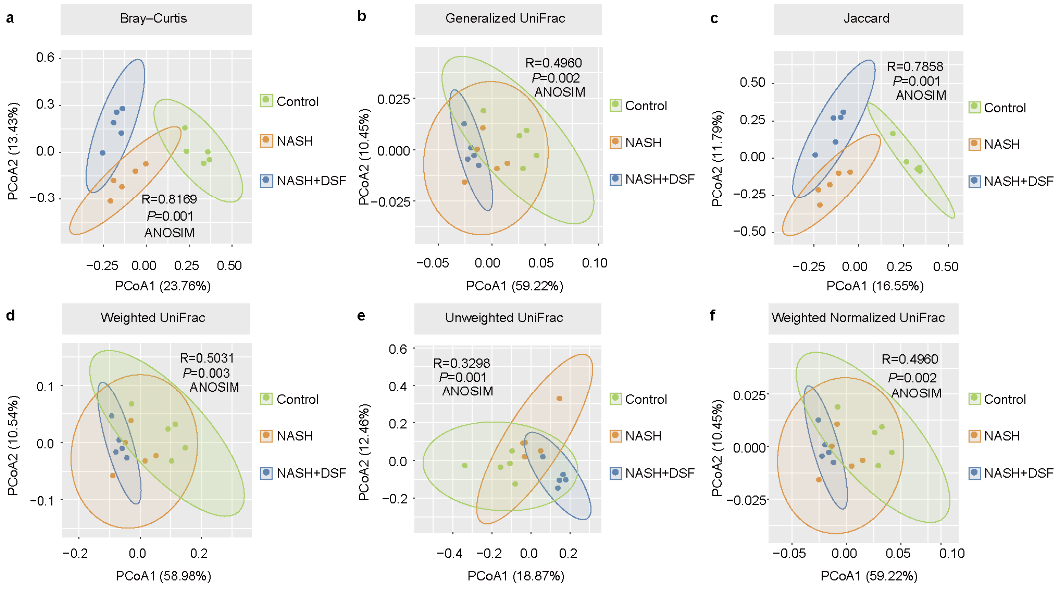

Supplementary Fig. 4. The gut microbiota from control, NASH and NASH+DSF mice were entirely separated. a-f 16S rRNA gene sequencing analysis in fecal bacterial DNA from control, NASH and NASH+DSF mice was performed. n = 5 individuals/group. Each point represented an individual mouse. PCoA analysis using Bray–Curtis (**a**), Generalized UniFrac (**b**), Jaccard (**c**), Weighted UniFrac (**d**), Unweighted UniFrac (**e**) and Weighted Normalized UniFrac (**f**) metric distances of beta diversity. Differences of data were calculated by ANOSIM test. Exact *P* values were all given. ANOSIM, analysis of similarities; DSF, disulfiram; NASH, nonalcoholic steatohepatitis; PCoA, principal coordinate analysis. Source data are provided as a Source Data file.

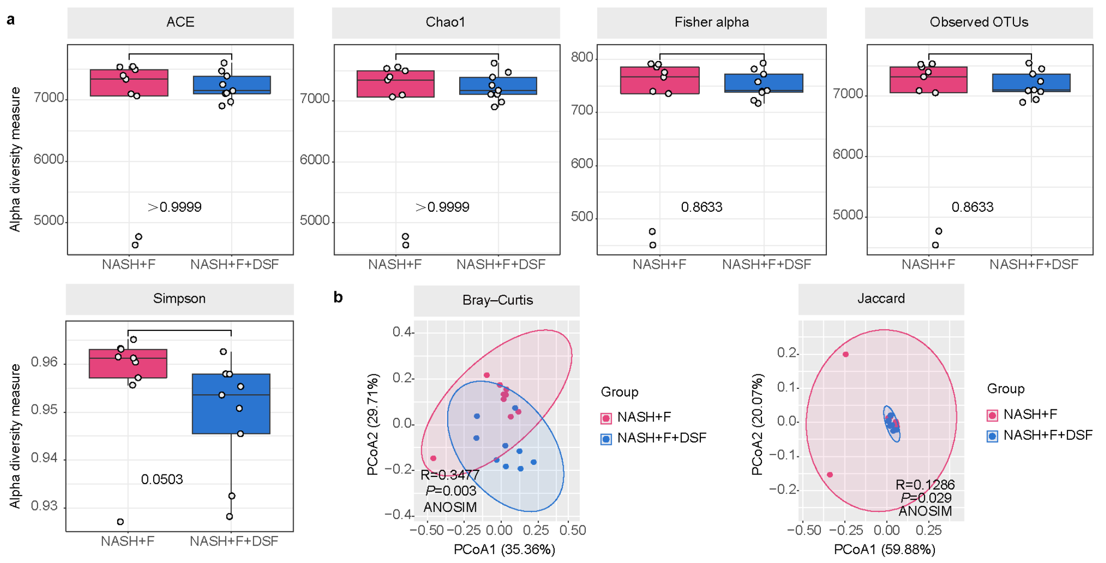

Supplementary Fig. 5. Alpha and beta diversities of gut microbiota among NASH+F and NASH+F+DSF mice. a Alpha diversity based on ACE, Chao1, Fisher alpha, Observed OTUs and Simpson indices. Differences of data were calculated by two-sided Mann-Whitney test. The horizontal bar within box represents median. The top and bottom of box represent 75th and 25th quartiles, respectively. The upper and lower whiskers extended 1.5 x the interquartile range from the upper edge and lower edge of the box represent maximum and minimum, respectively. **b** PCoA using Bray–Curtis and Jaccard metric distances of beta diversity. Differences of data were calculated by ANOSIM test. **a-b** n = 9 individuals/group. Each point represented an individual mouse. Exact *P* values were all given. ANOSIM, analysis of similarities; DSF, disulfiram; F, fecal samples obtained from patients with NASH; NASH, nonalcoholic steatohepatitis; PCoA, principal coordinate analysis. Source data are provided as a Source Data file.

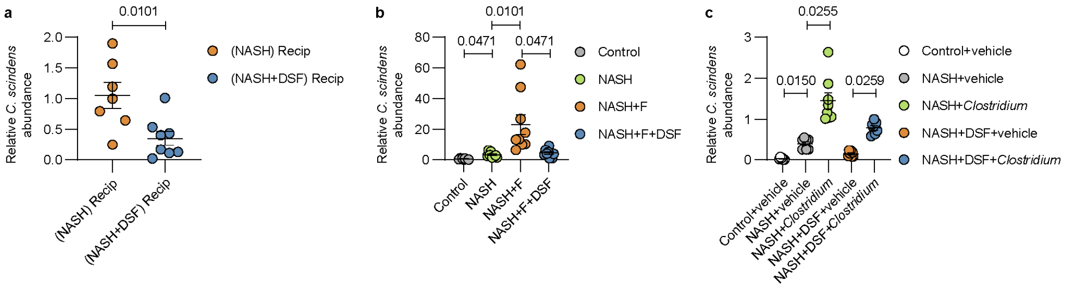

Supplementary Fig. 6. The abundance of *Clostridium* in FMT, HMA mice and bacterial colonization experiment. The relative *Clostridium* abundance in FMT, HMA mice and bacterial colonization experiment by qRT–PCR using primers specific for *C. scindens*. n = 7/8 individuals/group, respectively for FMT; n = 9 individuals/group for HMA mice; n = 8/8/8/8/7 individuals/group, respectively for bacterial colonization experiment. Each point represented an individual mouse. Differences of data were calculated by unpaired, two-sided t test (for 2 groups) or Kruskal-Wallis test (for more than 2 groups). Data were represented as mean ± SEM. Exact *P* values were all given. Data were pooled from three independent experiments. *C. scindens*, *Clostridium scindens*; DSF, disulfiram; F, fecal samples obtained from patients with NASH; NASH, nonalcoholic steatohepatitis. Source data are provided as a Source Data file.

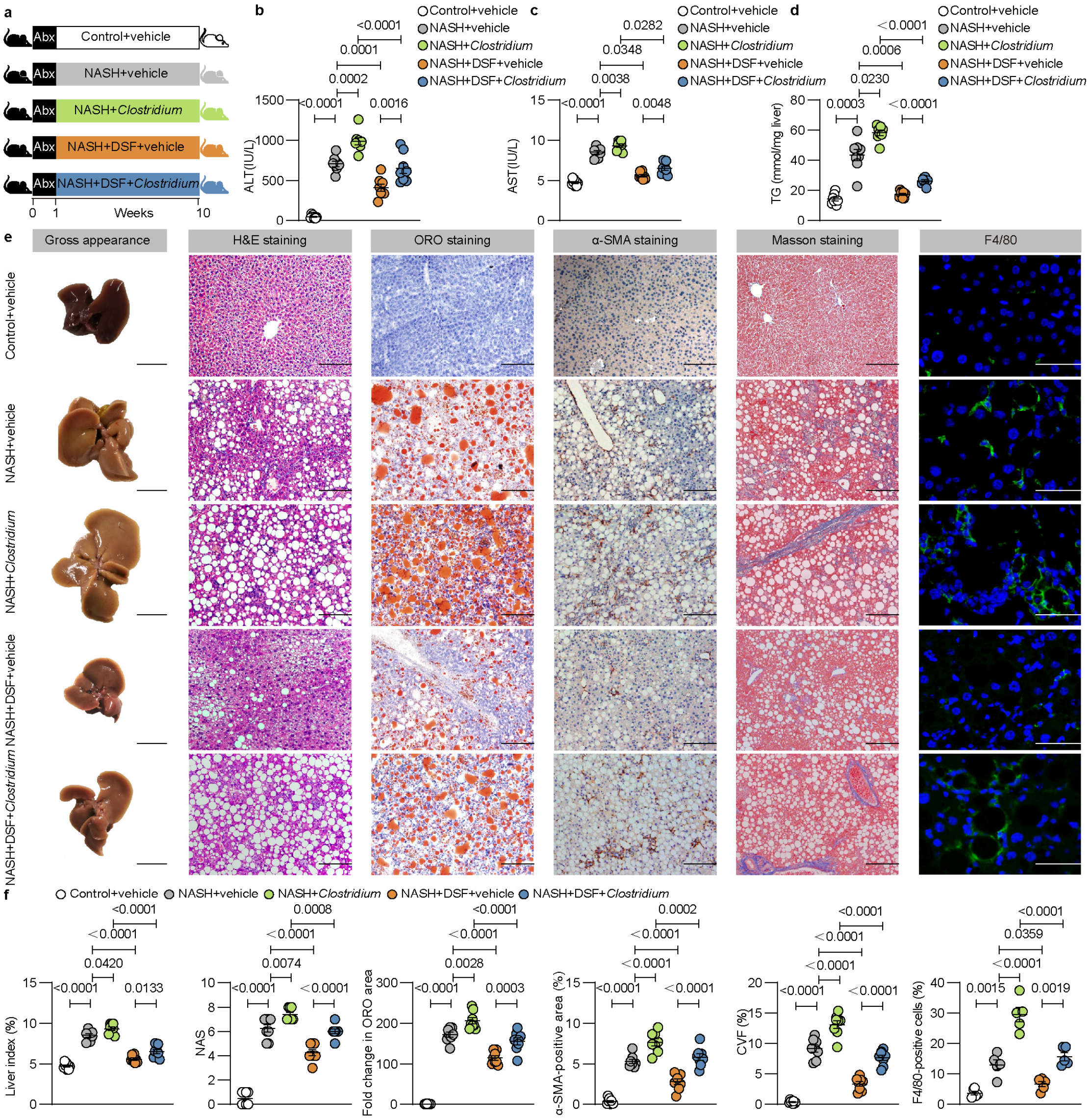

Supplementary Fig. 7. ***Clostridium*** **abolishes the ameliorating effects of DSF on NASH. a** Administration of *Clostridium* experimental design. WT mice were put on a course of intragastrically Abx administration for 1 week for gut microbiota depletion. Then mice were randomized into 5 groups (control+vehicle, NASH+vehicle, NASH+*Clostridium*, NASH+DSF+vehicle and NASH+DSF+*Clostridium*) under a control diet, CDAHFD, CDAHFD, CDAHFD+DSF and CDAHFD+DSF, respectively for 9 weeks. Meanwhile, mice were gavaged with *C. scindens* (3×10^{8} CFU/200 µL per mouse suspended in sterile 2107 medium) twice a week for 9 weeks. Equal sterile 2107 medium was used as vehicle. **b-d** Serum ALT and AST; hepatic TG. **e** Representative

images of gross appearance of the liver histology (1 cm) and photomicrographs of fixed liver sections after staining with H&E (200 μm), ORO (200 μm), α-SMA antibody (200 μm), Masson (200 μm) and F4/80 antibody (50 μm). **f** Quantification of the liver index (%), NAS, fold change in ORO area, α-SMA-positive area (%), CVF (%) and F4/80-positive cells (%). **a-f** n = 5 individuals/group for the F4/80 IF staining; n = 8 individuals/group for other experiments. Each point represented an individual mouse. Differences of data were calculated by Kruskal-Wallis test or ordinary one-way ANOVA depending on the sample distribution type. Data were represented as mean ± SEM. Exact *P* values were all given. Data were pooled from three independent experiments. Abx, antibiotic cocktail; α-SMA, α-smooth muscle actin; CVF, collagen volume fraction; DSF, disulfiram; H&E, hematoxylin and eosin; NAS, NAFLD activity score; NASH, nonalcoholic steatohepatitis; ORO, oil red O; TG, triglyceride. Source data are provided as a Source Data file.

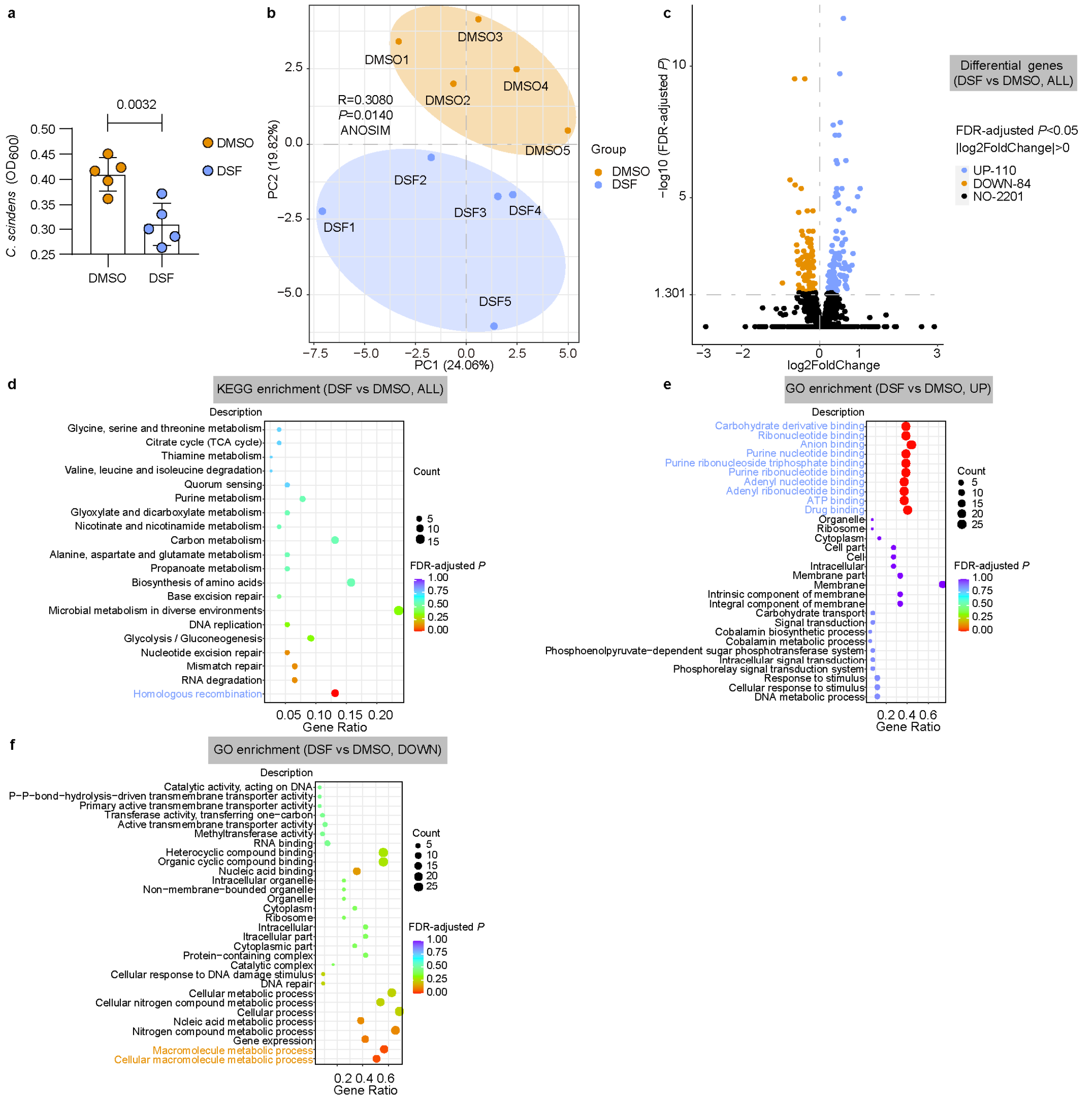

Supplementary Fig. 8. DSF directly inhibits the growth of *Clostridium*. a Growth of *C. scindens* with DMSO or DSF in a laboratory culture. n = 5 replicates/treatment. Data were expressed as mean ± SEM. Differences of data were assessed by unpaired, two-sided t test. Exact *P* value was provided. **b** PCA analysis on the gene expression value of all samples. Differences of data were assessed by ANOSIM test. **c** Volcano plot represented differential expression analysis of genes in DMSO group versus DSF group. Orange and blue points marked the genes with significantly decreased or increased expression respectively in DSF group compared to DMSO (FDR-adjusted $P < 0.05$). The x-axis showed log2FoldChange in expression and the y-axis represented the

significance level of the difference in gene expression (-log10 (FDR-adjusted $P < 0.05$)). **d** The most significant 20 KEGG pathways were selected and the Dotplot was employed. The x-axis showed the ratio of the number of differential genes annotated to the KEGG pathways to the total number of differential genes. The y-axis represented the KEGG pathways. **e**, **f** The most significant upregulated (**e**) or down-regulated (**f**) 30 GO terms were selected and the Dotplot was employed. The x-axis showed the ratio of the number of differential genes annotated to the GO terms to the total number of differential genes. The y-axis represented the GO terms. **d-f** The size of the dot represented the number of genes annotated to KEGG pathways/GO terms, and the color from red to purple represents the significance of the enrichment (FDR-adjusted $P <$ 0.05). *C. scindens*, *Clostridium scindens*; DMSO, dimethyl sulfoxide; DSF, disulfiram; GO, Gene Ontology; KEGG, Kyoto Encyclopedia of Genes and Genomes. Source data are provided as a Source Data file.

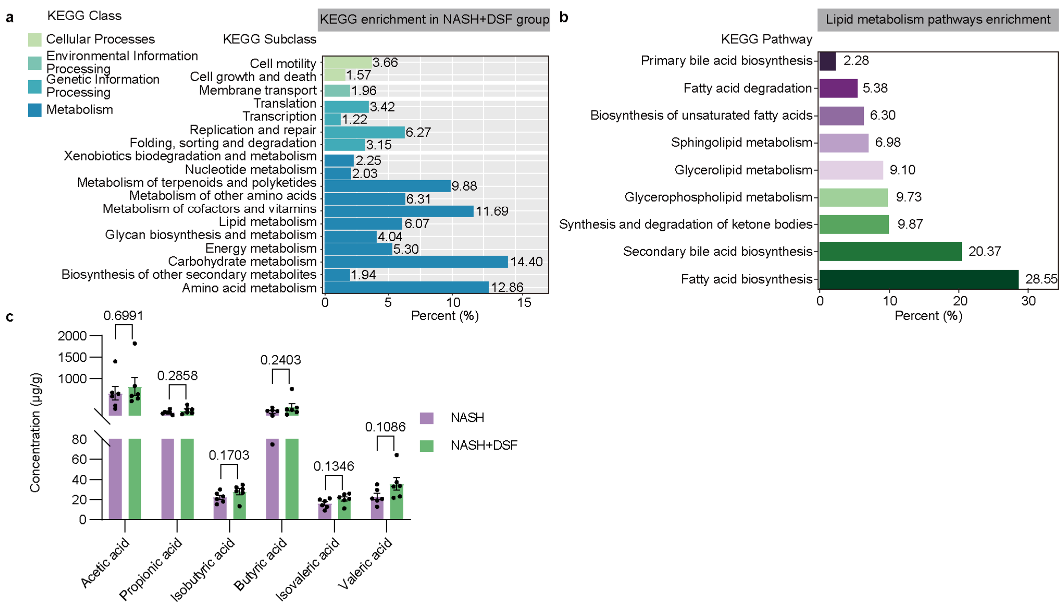

Supplementary Fig. 9. DSF treatment modulates secondary bile acid biosynthesis. **a, b** Analysis of 16S rRNA gene sequencing data from Fig. 3. n = 5 individuals. **a** Annotations of microbial gene functions of NASH+DSF mice on KEGG subclass enrichment. **b** Annotations of lipid metabolism pathway enrichment of NASH+DSF mice on KEGG pathway enrichment. **c** SCFAs concentration from cecum samples from NASH and NASH+DSF mice. n = 6 individuals/group. Each point represented an individual mouse. Differences of data were calculated by two-sided Mann-Whitney test or unpaired, two-sided t test depending on the sample distribution type. Data were represented as mean ± SEM. Exact *P* values were all given. DSF, disulfiram; KEGG, Kyoto Encyclopedia of Genes and Genomes; NASH, nonalcoholic steatohepatitis. Source data are provided as a Source Data file.

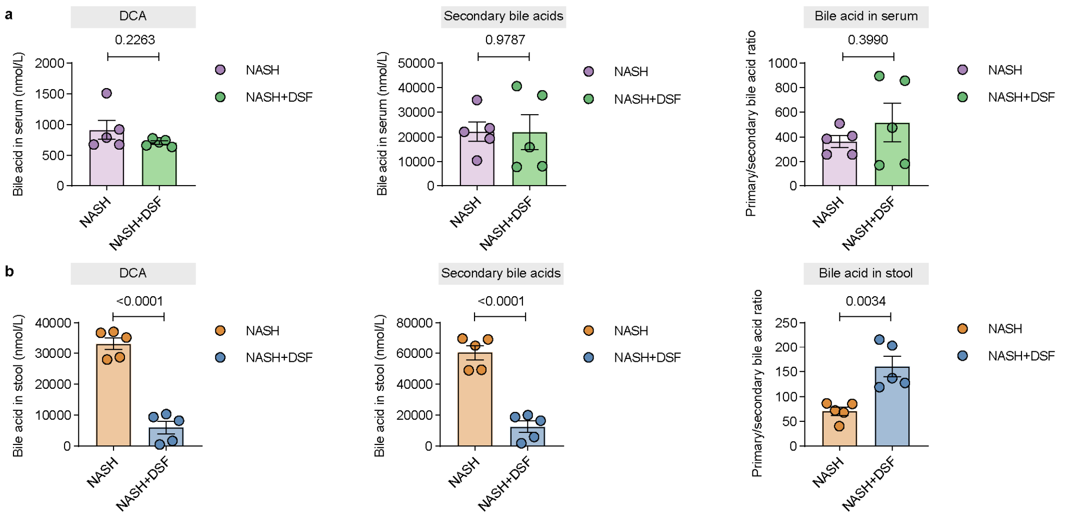

Supplementary Fig. 10. Bile acids in serum and stool of NASH and NASH+DSF mice. a The DCA level, secondary bile acids level and the primary/secondary bile acid ratio in serum of mice. **b** The DCA level, secondary bile acids level and the primary/secondary bile acid ratio in stool of mice. n = 5 individuals/group. Each point represented an individual mouse. Differences of data were calculated by two-sided Mann-Whitney test or unpaired, two-sided t test depending on the sample distribution type. Data were represented as mean ± SEM. Exact *P* values were all given. DCA, deoxycholic acid; DSF, disulfiram; NASH, nonalcoholic steatohepatitis. Source data are provided as a Source Data file.

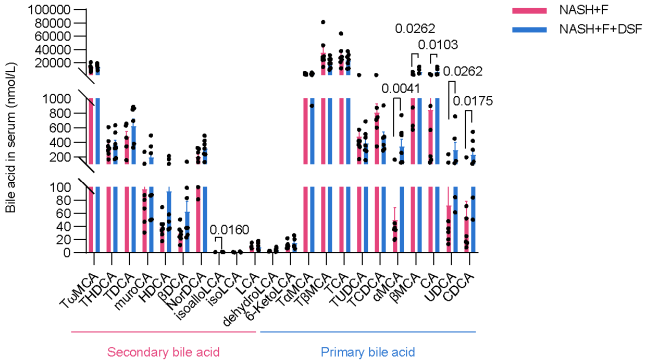

Supplementary Fig. 11. Bile acids in serum of NASH+F and NASH+F+DSF mice. n = 7 individuals/group. Each point represented an individual mouse. Differences of data were calculated by two-sided Mann-Whitney test or unpaired, two-sided t test depending on the sample distribution type. Data were represented as mean ± SEM. Exact *P* values were all given. DSF, disulfiram; F, fecal samples obtained from patients with NASH; NASH, nonalcoholic steatohepatitis. Source data are provided as a Source Data file.

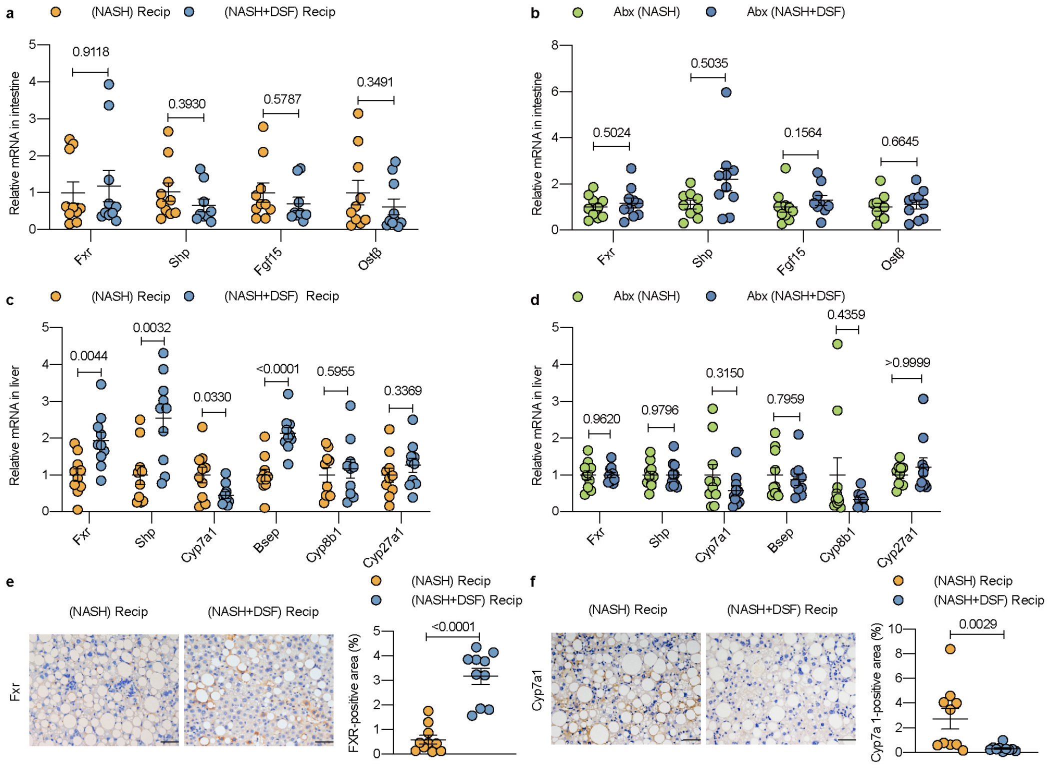

Supplementary Fig. 12. The gut microbiota is involved in DSF-induced hepatic FXR signaling activation. a The expressions of Fxr, Shp, Fgf15 and Ostβ in the intestines of NASH Recip and NASH+DSF Recip mice. **b** The expressions of Fxr, Shp, Fgf15 and Ostβ in the intestines of Abx (NASH) and Abx (NASH+DSF) mice. **c** The expressions of Fxr, Shp, Cyp7a1, Besp, Cyp8b1 and Cyp27a1 in the livers of NASH Recip and (NASH+DSF) Recip mice. **d** The expressions of Fxr, Shp, Cyp7a1, Besp, Cyp8b1 and Cyp27a1 in the livers of Abx (NASH) and Abx (NASH+DSF) mice. **e, f** IHC staining of Fxr and Cyp7a1 in the livers of NASH Recip and (NASH+DSF) Recip mice. **a-f** n = 10 individuals/group. Each point represented an individual mouse. Differences of data between 2 groups were calculated by two-sided Mann-Whitney test or unpaired, two-sided t test depending on the sample distribution type. Data were represented as mean ± SEM. Exact *P* values were all given. Scale bar, 50 μm. Data were pooled from three independent experiments. Abx, antibiotic cocktail; DSF, disulfiram; NASH, nonalcoholic steatohepatitis. Source data are provided as a Source Data file.

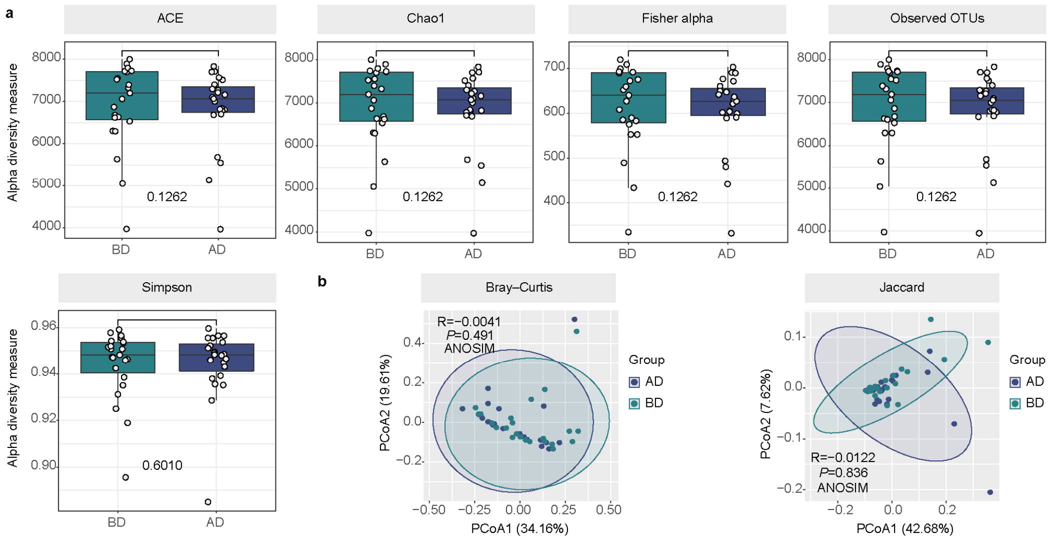

Supplementary Fig. 13. Alpha and beta diversities of gut microbiota among BD and AD groups. a Alpha diversity based on ACE, Chao1, Fisher alpha, Observed OTUs and Simpson indices. Differences of data were calculated by two-sided Wilcoxon matched-pairs signed rank test. The horizontal bar within box represents median. The top and bottom of box represent 75th and 25th quartiles, respectively. The upper and lower whiskers extended 1.5× the interquartile range from the upper edge and lower edge of the box represent maximum and minimum, respectively. **b** PCoA using Bray–Curtis and Jaccard metric distances of beta diversity. Differences of data were calculated by ANOSIM test. **a-b** n = 23 individuals/group. Each point represented an individual mouse. Exact *P* values were all given. AD, volunteers after DSF treatment; ANOSIM, analysis of similarities; BD, volunteers before DSF treatment; PCoA, principal coordinate analysis. Source data are provided as a Source Data file.

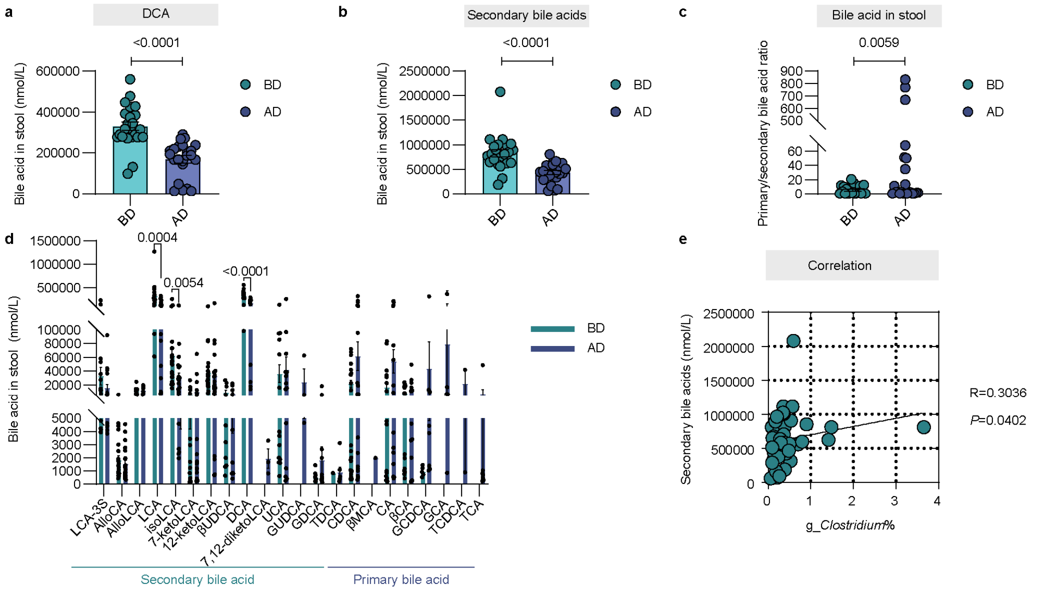

Supplementary Fig. 14. Bile acids in stool of BD and AD groups. a-d The DCA level, secondary bile acids level, the primary/secondary bile acid ratio and the bile acids profile in stool of BD and AD groups. **e** The correlation between the abundance of *Clostridium* and the secondary bile acids level was analyzed using Spearman's correlation (two-sided). 23 individuals each in BD and AD groups. Each point represented an individual. Differences of data between 2 groups were calculated by two-sided Wilcoxon matched-pairs signed rank test or two-sided paired t test depending on the sample distribution type. Data were represented as mean ± SEM. Exact *P* values were all given. AD, volunteers after DSF treatment; BD, volunteers before DSF treatment; DCA, deoxycholic acid. Source data are provided as a Source Data file.

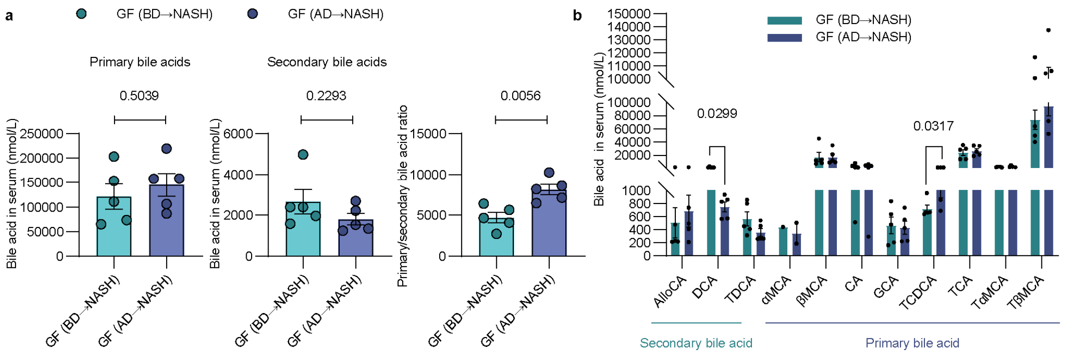

Supplementary Fig. 15. Bile acids in serum of GF mice. a, **b** The primary bile acids level, secondary bile acids level, primary/secondary bile acid ratio and bile acids profile in serum of GF mice. n = 5 individuals/group. Each point represented an individual. Differences of data between 2 groups were calculated by two-sided Mann-Whitney test or unpaired, two-sided t test depending on the sample distribution type. Data were represented as mean ± SEM. Exact *P* values were all given. AD, volunteers after DSF treatment; BD, volunteers before DSF treatment; GF, germ-free. Source data are provided as a Source Data file.

Supplementary Tables

Supplementary Table 1. Summary on statistics of the host properties and clinical indexes from volunteers

Classification	Index	Symbol	BD	AD	*P* value
Host property (Basic information)	Gender	Gender (F/M)	6/17	6/17	
Host property (Basic information)	Age (year)	Age	30.87±1.26	30.87±1.26	
Host property (Basic information)	Body mass index (kg/m^2)	BMI	23.49±0.61	23.39±0.56	0.2488
Host property (Basic information)	Height (m)	Height	1.71±0.01	1.71±0.01	0.3282
Host property (Basic information)	Weight (kg)	Weight	68.85±2.00	68.61±1.89	0.3112
Host property (Basic information)	Ethnicity (Han/other minorities)	Ethnicity (Han/other)	0/23	0/23	
Host property (Disease)	Hypertension	Hypertension (Y/N)	0/23	0/23	
Host property (Disease)	Diabetes	Diabetes (Y/N)	0/23	0/23	
Host property (Disease)	Chronic hepatitis	Chronic hepatitis	0/23	0/23	
Host property (Dietary)	Smoking	Smoking (Y/N)	4/23	4/23	
Host property (Dietary)	Alcohol	Alcohol (Y/N)	7/23	7/23	
Clinical index (Renal function)	Urea (mmol/L)	UREA	4.97±0.25	4.55±0.16	0.1223
Clinical index (Renal function)	Uric acid (μmol/L)	UA	365.8±20.37	341.3±25.07	0.1566
Clinical index (Liver function)	Alanine transaminase (U/L)	ALT	23.95±2.58	22.95±2.43	0.4103
Clinical index (Liver function)	Glutamic oxaloacetic transaminase (U/L)	AST	18.53±0.84	18.77±0.91	0.7115
Clinical index (Liver function)	Alkaline phosphatase (U/L)	ALP	63.14±2.93	63.04±2.72	0.9438
Clinical index (Liver function)	γ-glutamyl transpeptidase (U/L)	GGT	24.12±2.82	23.80±2.67	0.7592
Clinical index (Blood lipid)	Triglycerides (mmol/L)	TG	1.31±0.14	1.23±0.16	0.2659
Clinical index (Blood lipid)	Total cholesterol (mmol/L)	TCH	4.48±0.15	4.31±0.17	0.0562
Clinical index (Blood lipid)	High density lipoprotein (mmol/L)	HDL_C	1.30±0.05	1.23±0.05	0.0005
Clinical index (Blood lipid)	Low density lipoprotein (mmol/L)	LDL_C	2.52±0.13	2.32±0.14	0.0245
Clinical index (Blood routine)	White blood cell (10^9/L)	WBC	6.10±0.27	6.46±0.25	0.1191
Clinical index (Blood routine)	Red blood cells (10^{12}/L)	RBC	4.99±0.10	4.91±0.10	0.0586
Clinical index (Blood routine)	Platelet (10^9/L)	PLT	245.9±10.76	249.2±12.51	0.7002
Clinical index (Blood routine)	Mean corpuscular volume (fL)	MCV	88.92±1.09	88.03±1.07	0.0763
Clinical index (Blood routine)	Mean corpuscular hemoglobin (pg)	MCH	30.51±0.56	30.47±0.45	0.9450
Clinical index (Blood routine)	Hemoglobin (g/L)	HGB	149.6±2.54	149.0±2.74	0.6839
Clinical index (Fibrotouch)	Liver stiffness (KPA)	Liver stiffness	6.15±0.28	6.36±0.22	0.2973

Clinical index (Fibrotouch)	Fat attenuation (db/m)	Fat attenuation	267.1±8.75	263.7±9.05	0.4689

P value was determined by two-sided paired t test.

Data were represented as mean ± SEM.

n (BD) = 23 individuals; n (AD) = 23 individuals

Supplementary Table 2. Adverse events after DSF treatment

Systems	Symptoms	Day1	Day2	Day3	Day4	Day5	Day6	Day7	Occurrence rate (%)
Nervous-mental system	Depressive emotion	0	0	0	0	0	0	0	0.00
	Hallucination	0	0	0	0	0	0	0	0.00
	Headache	0	1	0	0	0	0	0	0.62
	Tinnitus	1	1	0	1	0	0	0	1.86
	Insomnia	0	0	0	0	0	0	0	0.00
	Drowsiness	1	0	0	0	0	0	0	0.62
Digestive system	Stomachache	0	0	0	0	0	0	0	0.00
	Anorexia	3	2	2	2	1	0	0	6.21
	Diarrhea	2	1	0	1	0	0	1	3.11
	Constipation	0	0	1	1	1	1	1	3.11
	Jaundice	0	0	0	0	0	0	0	0.00
	Bad breath	2	1	1	0	0	0	0	2.48
	Acid reflux	0	0	0	0	1	1	1	1.86
Other systems	Pruritus, rash, redness and swelling	0	0	0	0	0	0	0	0.00
	Vision changes	0	0	0	0	0	0	0	0.00
	Arthralgia	0	0	0	0	0	0	0	0.00

n = 23 individuals.

Supplementary Table 3. NAS criterion

Item	Score			
	0	1	2	3
Steatosis	< 5%	5%-33%	34%-66%	> 66%
Lobular inflammation (×200)	No foci	< 2 foci	2-4 foci	> 4 foci
Hepatocyte ballooning	None	Few	Many	

NAS with scores <3 indicating “no NASH”, ≥3 and <5 indicating “borderline NASH” and≥5 indicating “NASH”

Supplementary Table 4. Primers for qRT–PCR detection (SYBR Green).

Mouse gene	Forward (5'→3')	Reverse (5'→3')
β-actin	GGCGGACTGTTACTGAGCTG	CTGCGCAAGTTAGGTTTTGTCA
Tnf-α	GCAAACCAGACTTCTACTGCG	TTTGTATTGCTGGTTGCTGTG
Mcp-1	CTTCTGGGCCTGCTGTTCA	CCAGCCTACTCATTGGGATCA
Col1α1	GAGCGGAGAGTACTGGATCG	GCTTCTTTTCCTTGGGGTTC
Col1α2	CCTACATGGACCAGCAGACTG	GGAGGTCTTGGTGGTTTTGTA
Tgf-β1	CAACCCAGGTCCTTCCTAAA	GGAGAGCCCTGGATACCAAC
Fxr	TGGGCTCCGAATCCTCTTAGA	TGGTCCTCAAATAAGATCCTTGG
Shp	TCTGCAGGTCGTCCGACTATTC	AGGCAGTGGCTGTGAGATGC
Fgf15	ATGGCGAGAAAGTGGAACGG	GGACCAGCGGAGTACAGGT
Ostβ	AGATGCGGCTCCTTGGAATTA	TGGCTGCTTCTTTCGATTTCTG
Cyp7a1	TGGGCATCTCAAGCAAACAC	TCATTGCTTCAGGGCTCCTG
Bsep	GAGTGGTGGACAGAAGCAAA	TGAGGTAGCCATGTCCAGAA
Cyp8b1	GCCCTTACTCCAAATCCTACCA	TCGCACACATGGCTCGAT
Cyp27a1	GCACAGGAGAGTACGGAGG	CGGGCAAGTGCAGCACATA
