# Supplementary material for: Disulfiram ameliorates nonalcoholic steatohepatitis by modulating the gut microbiota and bile acid metabolism
Source: Nat Commun. 2022 Nov 11;13:6862. doi: 10.1038/s41467-022-34671-1 (PMC9651870; doi:10.1038/s41467-022-34671-1)
Supplement: Supplementary file 2 — Reporting Summary [file 41467_2022_34671_MOESM2_ESM.pdf]

nature portfolio

Corresponding author(s): Hong Wei. Shiming Yang and Bo Tang

Last updated by author(s): Oct 25, 2022

Reporting Summary

Nature Portfolio wishes to improve the reproducibility of the work that we publish. This form provides structure for consistency and transparency in reporting. For further information on Nature Portfolio policies, see our Editorial Policies and the Editorial Policy Checklist.

Statistics

For all statistical analyses, confirm that the following items are present in the figure legend, table legend, main text, or Methods section.

n/a	Confirmed	
☐	☒	The exact sample size (*n*) for each experimental group/condition, given as a discrete number and unit of measurement
☐	☒	A statement on whether measurements were taken from distinct samples or whether the same sample was measured repeatedly
☐	☒	The statistical test(s) used AND whether they are one- or two-sided<br>*Only common tests should be described solely by name; describe more complex techniques in the Methods section.*
☐	☒	A description of all covariates tested
☐	☒	A description of any assumptions or corrections, such as tests of normality and adjustment for multiple comparisons
☐	☒	A full description of the statistical parameters including central tendency (e.g. means) or other basic estimates (e.g. regression coefficient) AND variation (e.g. standard deviation) or associated estimates of uncertainty (e.g. confidence intervals)
☐	☒	For null hypothesis testing, the test statistic (e.g. *F*, *t*, *r*) with confidence intervals, effect sizes, degrees of freedom and *P* value noted<br>*Give P values as exact values whenever suitable.*
☒	☐	For Bayesian analysis, information on the choice of priors and Markov chain Monte Carlo settings
☒	☐	For hierarchical and complex designs, identification of the appropriate level for tests and full reporting of outcomes
☐	☒	Estimates of effect sizes (e.g. Cohen's *d*, Pearson's *r*), indicating how they were calculated

Our web collection on statistics for biologists contains articles on many of the points above.

Software and code

Policy information about availability of computer code

Data collection	The comparative transcriptomes, metagenomic and 16S rRNA gene sequencing data were collected by Illumina NovaSeq 6000. Bile Acids data were collected by UPLC-MS/MS (ACQUITY UPLC-Xevo TQ-S). Short-chain fatty acids data were collected by gas chromatography-mass spectrometry (GC-MS) system (Trace 1300 and ISQ 7000, Thermo Scientific). Images of liver histology were taken with an upright fluorescence microscope (Olympus, BX63, Japan). Schematic diagram of experimental designs and were drawn with Adobe illustrator 2022. Other data were collected with GraphPad Prism software (v8) and Microsoft Excel 2019.
Data analysis	Heatmap was generated by STAMP (v2). Statistical tests were analyzed and charts were generated using GraphPad Prism software (v8). Liver histology quantification was performed using ImageJ software (v1.8.0). KneadData (v0.7.4), Kraken2 (v2.1.1) and Bracken (v2.6.0) software were used to analyze metagenomic sequencing data.

For manuscripts utilizing custom algorithms or software that are central to the research but not yet described in published literature, software must be made available to editors and reviewers. We strongly encourage code deposition in a community repository (e.g. GitHub). See the Nature Portfolio guidelines for submitting code & software for further information.

Data

Policy information about availability of data

All manuscripts must include a data availability statement. This statement should provide the following information, where applicable:

- Accession codes, unique identifiers, or web links for publicly available datasets
- A description of any restrictions on data availability
- For clinical datasets or third party data, please ensure that the statement adheres to our policy

The comparative transcriptomes, metagenomic and 16S rRNA gene sequencing data generated in this study have been deposited in the European Nucleotide Archive database under accession code PRJEB49623 [https://www.ebi.ac.uk/ena/browser/view/PRJEB49623]. The raw healthy volunteer-related clinical trial raw data, which could compromise protection of privacy of research participants are protected and are not available due to data privacy laws. The data supporting the findings of this study generated in this study are provided in the Supplementary Information and Source Data file. Source data are provided as a Source Data file. Source data are provided with this paper.

Human research participants

Policy information about studies involving human research participants and Sex and Gender in Research.

Reporting on sex and gender	Sex and/or gender were considered in the study design. Sex and/or gender of participants was determined based on self-report. Data was reported disaggregated for sex and gender where this information has been collected and consent has been obtained for reporting and sharing individual-level data; 6 females and 17 males were recruited. We did not conduct post hoc sex- and gender-based analysis.
Population characteristics	23 healthy volunteers (6 females and 17 males) aged 18-59 years who meet the following exclusion criteria were recruited recruited from the Department of Gastroenterology in the Second Affiliated Hospital of Army Medical University in this study (1: Patients with alcoholic fatty liver disease; 2: Patients with severe renal dysfunction; 3: According to the judgment of the investigators, patients with any clinically significant medical conditions that may affect research participation and/or personal health; 4: Those who have recently taken a variety of drugs for the treatment of diseases; 5: Those who have recently consumed any alcoholic food or used any alcoholic products; 6: Those who are pregnant or breastfeeding; 7: Those who are participated in a weight loss plan within the past 3 months; 8: Those with a weight change of ≥5%; 9: Those who have donated blood within 3 months or plan to donate blood during the study period; 10: Those who have not signed the informed consent form and or could not complete the research process).
Recruitment	23 healthy volunteers (6 females and 17 males) aged 18-59 years were enrolled by the researchers. All of the participants provided written informed consent. The exclusion criteria were listed as follows (1: Patients with alcoholic fatty liver disease; 2: Patients with severe renal dysfunction; 3: According to the judgment of the investigators, patients with any clinically significant medical conditions that may affect research participation and/or personal health; 4: Those who have recently taken a variety of drugs for the treatment of diseases; 5: Those who have recently consumed any alcoholic food or used any alcoholic products; 6: Those who are pregnant or breastfeeding; 7: Those who are participated in a weight loss plan within the past 3 months; 8: Those with a weight change of ≥5%; 9: Those who have donated blood within 3 months or plan to donate blood during the study period; 10: Those who have not signed the informed consent form and or could not complete the research process).
Ethics oversight	The research was approved by the Medical Ethics Committee of the Second Affiliated Hospital of Army Medical University (Chongqing, China) (Approved No. 2021-055-01) and was registered with the Chinese Clinical Trial Registry (ChiCTR) in the WHO Registry Network (Registration number: ChiCTR2100048035). All volunteers provided written informed consent before participated in this study.

Note that full information on the approval of the study protocol must also be provided in the manuscript.

Field-specific reporting

Please select the one below that is the best fit for your research. If you are not sure, read the appropriate sections before making your selection.

☒ Life sciences ☐ Behavioural & social sciences ☐ Ecological, evolutionary & environmental sciences

For a reference copy of the document with all sections, see nature.com/documents/nr-reporting-summary-flat.pdf

Life sciences study design

All studies must disclose on these points even when the disclosure is negative.

Sample size	Animals' number calculation was by "resource equation" method. According to this method, a value "E" is measured. The value of E should lie between 10 and 20. If E is less than 10 then adding more animals will increase the chance of getting more significant result, but if it is more than 20 then adding more animals will not increase the chance of getting significant results. It is considerable to be applicable to all animal experiments. Any sample size, which keeps E between 10 and 20 should be considered as an adequate. E can be measured by following formula: E = Total number of animals − Total number of groups).
Data exclusions	No data were excluded.
Replication	All experimental findings were pooled from three independent experiments.
Randomization	Mice were strictly randomized into groups.
Blinding	The investigators were blinded to group allocation during data collection and analysis.

Reporting for specific materials, systems and methods

We require information from authors about some types of materials, experimental systems and methods used in many studies. Here, indicate whether each material, system or method listed is relevant to your study. If you are not sure if a list item applies to your research, read the appropriate section before selecting a response.

Materials & experimental systems

n/a	Involved in the study
☐	☒ Antibodies
☒	☐ Eukaryotic cell lines
☒	☐ Palaeontology and archaeology
☐	☒ Animals and other organisms
☐	☒ Clinical data
☒	☐ Dual use research of concern

Methods

n/a	Involved in the study
☒	☐ ChIP-seq
☒	☐ Flow cytometry
☒	☐ MRI-based neuroimaging

Antibodies

Antibodies used	IHC staining was performed using the following antibodies: α-SMA polyclonal antibody (1:1000; Cat# 14395-1-AP, Proteintech, Rosemont, USA), FXR (1:200; Cat# sc-25309, Santa Cruz Biotechnology, Santa Cruz, USA) and anti-CYP7A1 (1:100; Cat# sc-518007, Santa Cruz Biotechnology). IF staining was performed using anti-F4/80 rabbit polyclonal antibody (1:500, Cat# GB11027, Servicebio, Wuhan, China).
Validation	α-SMA polyclonal antibody (Tested Reactivity: Human, Mouse, Rat; Applications: WB, IP, IHC, IF, FC, competitive binding assay, ELISA). FXR (Tested Reactivity: mouse, rat and human; Applications: WB, IP, IF and ELISA). anti-CYP7A1 (Tested Reactivity: mouse, rat and human; Applications: WB, IP, IF and ELISA). anti-F4/80 rabbit polyclonal antibody (Tested Reactivity: Mouse; Applications: IHC/IF).

Animals and other research organisms

Policy information about studies involving animals; ARRIVE guidelines recommended for reporting animal research, and Sex and Gender in Research

Laboratory animals	C57BL/6 WT mice were purchased from Vital River Laboratories (Beijing, China). Fxr-/- mice with a C57BL/6 background were obtained from the Jackson Laboratory (Cat# 007214, Bar Harbor, USA). Prof. WH from the Department of Laboratory Animal Science (Army Medical University) provided the GF mice. Male mice aged 6 to 8 weeks at the beginning of the study were used.
Wild animals	The study didn't involve wild animals.
Reporting on sex	Only male mice were considered in this study.
Field-collected samples	The study didn't involve samples collected from fields.
Ethics oversight	The animal protocols were approved by the Laboratory Animal Welfare and Ethics Committee and adhered to the Animal Ethics Statement (Approved No. AMUWEC20210143) (Army Medical University).

Note that full information on the approval of the study protocol must also be provided in the manuscript.

Clinical data

Policy information about clinical studies

All manuscripts should comply with the ICMJE guidelines for publication of clinical research and a completed CONSORT checklist must be included with all submissions.

Clinical trial registration	the Chinese Clinical Trial Registry (ChiCTR) in the WHO Registry Network (Registration number: ChiCTR2100048035).
Study protocol	The clinical trial protocol was provided.
Data collection	The first volunteer was included on the 08/01/2021, the last experiment was completed on the 09/01/2021 and the study was finished on the 11/01/2021 after statistical analysis. The height and weight were measured, and the body mass index (BMI) was calculated. Human serum and fecal samples were obtained from all subjects who received oral DSF (250 mg qd, Mitsubishi Tanabe Pharma Corporation, Osaka, Japan) for 7 days. Blood samples were obtained after an overnight fast of 10 h, and serum was obtained by centrifugation (3000 x g, 10 min). The fecal samples were stored at −80 °C. Clinical parameters were determined in the laboratory department of the Second Affiliated Hospital of Army Medical University. Liver stiffness and hepatic fat attenuation were confirmed by a noninvasive, integrated image-guided detection system (the FibroTouch system) to assess the liver fibrosis and fatty liver levels. Adverse reactions were collected that occurred during the 7-day follow-up period. Metagenomic Sequencing was performed at Novogene in Beijing, China. Bile acid analysis was performed by Metabo-Profile Inc. in Shanghai, China. Other studies were performed in the Second Affiliated Hospital of Army Medical University in Chongqing, China.
Outcomes	1. To describe the diversity, composition and functional profile of gut microbiota before and after 7 days' DSF treatment.<br>Human serum and fecal samples were obtained from all subjects who received oral DSF (250 mg qd, Mitsubishi Tanabe Pharma Corporation, Osaka, Japan) for 7 days. Blood samples were obtained after an overnight fast of 10 h, and serum was obtained by centrifugation (3000 x g, 10 min). The fecal samples were stored at −80 °C and used for metagenomic sequencing (Illumina NovaSeq 6000 platform, Novogene) which assess the diversity, composition of gut microbiota before and after 7 days' DSF treatment. The serum samples are used for functional profile (bile acid analysis) of gut microbiota before and after 7 days' DSF treatment (UPLC-MS/MS, ACQUITY UPLC-Xevo TQ-S, Metabo-Profile Inc.).<br>2. To describe the changes in renal function, liver function, blood lipid, blood routine, etc. before and after 7 days' DSF treatment.<br>Human serum samples were obtained from all subjects who received oral DSF (250 mg qd, Mitsubishi Tanabe Pharma Corporation, Osaka, Japan) for 7 days. Blood samples were obtained after an overnight fast of 10 h, and serum was obtained by centrifugation (3000 x g, 10 min). Renal function, liver function, blood lipid, blood routine, etc. before and after 7 days' DSF treatment were determined in the laboratory department of the Second Affiliated Hospital of Army Medical University. Liver stiffness and hepatic fat attenuation were confirmed by a noninvasive, integrated image-guided detection system (the FibroTouch system) to assess the liver fibrosis and fatty liver levels.
